# Unraveling *Mycobacterium tuberculosis* genomic diversity and evolution in Lisbon, Portugal, a highly drug resistant setting

**DOI:** 10.1186/1471-2164-15-991

**Published:** 2014-11-18

**Authors:** João Perdigão, Hugo Silva, Diana Machado, Rita Macedo, Fernando Maltez, Carla Silva, Luisa Jordao, Isabel Couto, Kim Mallard, Francesc Coll, Grant A Hill-Cawthorne, Ruth McNerney, Arnab Pain, Taane G Clark, Miguel Viveiros, Isabel Portugal

**Affiliations:** Centro de Patogénese Molecular, URIA, Faculdade de Farmácia da Universidade de Lisboa, Av. Prof. Gama Pinto, 1649-003 Lisboa, Portugal; Grupo de Micobactérias, Unidade de Microbiologia Médica, Instituto de Higiene e Medicina Tropical, Universidade Nova de Lisboa (IHMT/UNL), Lisboa, Portugal; Public Health Department, Public Health Laboratory: Mycobacteriology/Tuberculosis, Administração Regional de Saúde de Lisboa e Vale do Tejo, I.P, Lisboa, Portugal; Serviço de Infecciologia, Hospital de Curry Cabral, Lisboa, Portugal; Departamento de Doenças Infecciosas, Instituto Nacional de Saúde Dr. Ricardo Jorge, Lisboa, Portugal; Centro de Recursos Microbiológicos (CREM), Faculdade de Ciências e Tecnologia, Universidade Nova de Lisboa, Caparica, Lisboa, Portugal; Faculty of Infectious and Tropical Diseases, London School of Hygiene & Tropical Medicine, Keppel Street, London, WC1E 7HT UK; Pathogen Genomics Laboratory, King Abdullah University of Science and Technology (KAUST), Thuwal, Makkah, Kingdom of Saudi Arabia; Sydney Emerging Infections and Biosecurity Institute and School of Public Health, Sydney Medical School, University of Sydney, Sydney, NSW 2006 Australia

**Keywords:** Whole genome sequencing, MDR-TB, XDR-TB, Lisboa family, Microevolution

## Abstract

**Background:**

Multidrug- (MDR) and extensively drug resistant (XDR) tuberculosis (TB) presents a challenge to disease control and elimination goals. In Lisbon, Portugal, specific and successful XDR-TB strains have been found in circulation for almost two decades.

**Results:**

In the present study we have genotyped and sequenced the genomes of 56 *Mycobacterium tuberculosis* isolates recovered mostly from Lisbon. The genotyping data revealed three major clusters associated with MDR-TB, two of which are associated with XDR-TB. Whilst the genomic data contributed to elucidate the phylogenetic positioning of circulating MDR-TB strains, showing a high predominance of a single SNP cluster group 5. Furthermore, a genome-wide phylogeny analysis from these strains, together with 19 publicly available genomes of *Mycobacterium tuberculosis* clinical isolates, revealed two major clades responsible for M/XDR-TB in the region: Lisboa3 and Q1 (LAM).

The data presented by this study yielded insights on microevolution and identification of novel compensatory mutations associated with rifampicin resistance in *rpoB* and *rpoC*. The screening for other structural variations revealed putative clade-defining variants. One deletion in PPE41, found among Lisboa3 isolates, is proposed to contribute to immune evasion and as a selective advantage. Insertion sequence (IS) mapping has also demonstrated the role of IS*6110* as a major driver in mycobacterial evolution by affecting gene integrity and regulation.

**Conclusions:**

Globally, this study contributes with novel genome-wide phylogenetic data and has led to the identification of new genomic variants that support the notion of a growing genomic diversity facing both setting and host adaptation.

**Electronic supplementary material:**

The online version of this article (doi:10.1186/1471-2164-15-991) contains supplementary material, which is available to authorized users.

## Background

Tuberculosis (TB) is responsible for approximately 1.4 million deaths each year and is considered a Global Health Emergency by the World Health Organization (WHO). Portugal is the Western European country that over the last few decades has had one of the highest TB notification rates in Europe (24.7 cases per 100 000) [1]. Although this rate is considered intermediate, the difficulty is the growing threat of drug resistance. In particular, the two most difficult-to-treat forms: multidrug-resistance (MDR, resistance to the two most powerful first-line drugs – isoniazid (INH), and rifampicin (RIF)) and extensive drug resistance (XDR, MDR plus resistance to fluoroquinolones (FQ), and a second-line injectable drug) [2, 3].

The TB situation in the capital city, Lisbon (incidence 31.5 cases / 100 000 in 2010) has been extensively studied [4–8]. Laboratory data on resistance prevalence point to high XDR-TB rates in the region, which in recent years have ranged between 44.3-66.1% of the MDR-TB clinical isolates [9].Genotyping studies using IS*6110* Restriction Fragment Length Polymorphism (RFLP), Spoligotyping, and more recently, through the characterization of Mycobacterial Interspersed Repetitive Units – Variable Number of Tandem Repeats (MIRU-VNTR), have led to the identification of a family of close genetic clusters in the 90’s: the Lisboa family, highly associated with MDR- and now XDR-TB [4, 7, 8].The Lisboa family has been defined a group of strains/clusters sharing a similar RFLP-IS*6110* profile (nine bands), belonging to the LAM lineage and/or sharing a similarity rate of at least 95% when genotyped by 12-*loci* MIRU-VNTR [7, 9]. The prevalence of this family in the region may account to up to 74.0% and 80.0% of MDR- and XDR-TB cases, respectively [4, 5, 9].

Another genetically close and endemic cluster, also belonging to the LAM lineage, named Q1 also plays an important role in MDR- and XDR-TB in the region and its impact on public health and drug-resistant TB in the region has been addressed in previous publications [5, 9]. When genotyped by 12-*loci* MIRU-VNTR, the Q1 cluster strains have been shown to share 11 MIRU-VNTR loci alleles with the most important Lisboa cluster (Lisboa3) but yet, bearing distinct mutational profiles on *rpsL*, *rrs* and *gyrA* genes [5]. A characteristic deletion of spacers 38–43 in the Direct Repeat *locus* has also been observed alongside with a characteristic spoligotyping LAM signature (data not published). Recently, mutation A80P in the *gidB* gene, responsible for low-level streptomycin (STP) resistance, has been proposed as a marker for Q1 strains [10].

The etiologic agents of TB are the bacterial (sub)species belonging to *Mycobacterium tuberculosis* complex (MTC), such as *Mycobacterium tuberculosis sensu stricto* (*M. tuberculosis*) or *Mycobacterium bovis*[11, 12]. *M. tuberculosis* has been regarded for many years as a genetically monomorphic pathogen. Nevertheless, the high-throughput genomic sequencing of diverse clinical strains has revealed a higher degree of variation than initially anticipated [13–16]. Next-Generation Sequencing (NGS) technology is allowing new insights on the mode of transmission and evolution of the MTC [17, 18]. Furthermore, the ability to compare, at the genomic level, identical strains in different stages of resistance acquisition can also provide new data on the genomic adaptation and compensation to the fixation of resistance-associated mutations in the host’s bacilli population [18, 19].

In this regard, the genomic determinants of the Lisboa family and Q1 strains are yet to be characterized. In the present study, we have genotyped and sequenced the genomes of 56 *M. tuberculosis* clinical isolates (sourced from the Lisbon Health Region) with the aim of gaining insights into the genomic diversity and microevolution of prevalent MDR- and XDR-TB circulating strains in the Lisbon region.

## Results

Of 56 *M. tuberculosis* isolates studied, 36 (64.3%) were resistant to INH and RIF and were therefore classified as MDR-TB isolates, of which we were able to determine the resistance to second-line drugs for 24 isolates. In total, 10 MDR-TB isolates were also classified as XDR-TB (Table 1).

**Table 1:** **Isolate characteristics: DST and data derived from WGS including mapping indicators**

Isolate	DST ^a^		SCG	PGG	Spoligotype ^b^		SNPs								INDELs ^c^		Mapping Indicators ^d^	
	First-Line	Second-Line			SIT	Clade	Non-synonymous mutations (N _s_ )	Synonymous mutations (S)	Total in Coding Regions (T _c_ )	Total in Non-Coding Regions	Total	N _s_ /S Ratio	T _v_ /T _s_ Ratio	T _c_ /Total	Total	Size Range	Mean Read Depth	Coverage (%)
ARS10348	IRS	ETH	5	2	20	LAM1	410	296	706	243	949	1.3851	0.5865	0.7439	96	1-24	135.18	98.86
ARS11131	IRSP	CAP AMK OFX MOX ETH	5	2	1106	LAM4	381	286	667	237	904	1.3322	0.6308	0.7378	95	1-24	101.56	98.89
ARS11285	IRS	AMK OFX MOX	4	2	119	X1	429	302	731	283	1014	1.4205	0.6031	0.7209	114	1-37	159.38	99.83
ARS11463	I	nd	5	2	64	LAM6	382	255	637	241	878	1.4980	0.5575	0.7255	76	1-24	52.42	99.47
ARS11661	IS	nd	5	2	1106	LAM4	378	284	662	234	896	1.3310	0.6443	0.7388	96	1-24	110.16	98.84
ARS12740	IRSP	ETH	5	2	1106	LAM4	392	287	679	239	918	1.3659	0.6456	0.7397	95	1-24	97.66	98.81
ARS1717	IRP	OFX ETH	6a	3	2258	Unknown	263	158	421	160	581	1.6646	0.5651	0.7246	67	1-27	68.89	99.76
ARS1760	I	nd	5	2	64	LAM6	379	262	641	242	883	1.4466	0.5725	0.7259	83	1-53	69.23	99.40
ARS1900	IRSEP	CAP KAN OFX ETH	5	2	20	LAM1	414	301	715	250	965	1.3754	0.6009	0.7409	101	1-24	148.85	98.75
ARS1930	IRSP	na	5	2	42	LAM9	389	268	657	228	885	1.4515	0.6158	0.7424	90	1-52	90.43	98.81
ARS2061	IRP	CAP AMK KAN OFX ETH CS PAS	5	2	1106	LAM4	379	283	662	239	901	1.3392	0.6346	0.7347	89	1-24	80.51	98.83
ARS2202	IRSP	OFX ETH CS	5	2	20	LAM1	404	284	688	243	931	1.4225	0.5889	0.7390	91	1-24	79.46	98.99
ARS2573	I	nd	5	2	20	LAM1	400	277	677	231	908	1.4440	0.6250	0.7456	97	1-49	82.71	99.03
ARS3649	IRSEP	KAN OFX ETH	5	2	20	LAM1	399	282	681	242	923	1.4149	0.5814	0.7378	91	1-24	72.67	98.92
ARS3806	I	nd	5	2	2535	Unknown	386	283	669	233	902	1.3640	0.6277	0.7417	85	1-24	195.56	98.84
ARS4857	IRP	na	5	2	1106	LAM4	381	279	660	234	894	1.3656	0.6418	0.7383	92	1-24	91.12	98.86
ARS5858	IREP	OFX	5	2	20	LAM1	395	284	679	243	922	1.3908	0.6401	0.7364	102	1-24	142.10	98.98
ARS6483	IRSEP	OFX ETH	5	2	20	LAM1	406	303	709	246	955	1.3399	0.5839	0.7424	98	1-24	92.22	98.79
ARS6539	IS	nd	5	2	20	LAM1	407	303	710	249	959	1.3432	0.5933	0.7404	63	1-24	273.98	98.87
ARS6559	I	nd	5	2	81	LAM9	394	292	686	233	919	1.3493	0.5953	0.7465	86	1-24	63.55	98.76
ARS7496	IS	nd	2	1	1	Beijing	589	410	999	405	1404	1.4366	0.6404	0.7115	175	1-39	109.62	99.30
ARS7571	I	nd	5	2	211	LAM3	388	265	653	244	897	1.4642	0.5749	0.7280	93	1-24	123.02	99.48
ARS7860	IS	nd	5	2	811	LAM4	378	279	657	232	889	1.3548	0.6318	0.7390	86	1-24	72.59	98.60
ARS7884	IRSEP	OFX ETH	5	2	20	LAM1	409	300	709	244	953	1.3633	0.5874	0.7440	100	1-24	177.31	98.81
ARS8437	IRSP	CAP ETH	5	2	20	LAM1	405	296	701	247	948	1.3682	0.6005	0.7395	92	1-24	179.07	98.78
ARS8600	I	nd	5	2	20	LAM1	407	291	698	238	936	1.3986	0.6262	0.7457	74	1-49	254.44	98.97
ARS9427	I	nd	3a	1	26	CAS1-Delhi	632	411	1043	422	1465	1.5377	0.5773	0.7119	152	1-36	44.07	99.82
FF181_97	IRS	na	5	2	20	LAM1	417	300	717	249	966	1.3900	0.6185	0.7422	24	1-9	772.29	98.99
FF291_98	IRS	na	5	2	20	LAM1	416	294	710	250	960	1.4150	0.6063	0.7396	31	1-18	658.49	98.97
FF359_98	IRS	na	5	2	20	LAM1	419	293	712	254	966	1.4300	0.5946	0.7371	31	1-18	836.93	99.01
FF674_96	Susceptible	na	4	2	91	X3	428	323	751	276	1027	1.3251	0.6047	0.7313	34	1-33	961.69	99.76
HCC1095_10	IRE	nd	3b	2	53	T1	429	314	743	292	1035	1.3662	0.5577	0.7179	119	1-24	185.45	99.61
HCC1276_11	IRSEP	CAP AMK OFX MOX ETH	5	2	1106	LAM4	392	281	673	243	916	1.3950	0.6350	0.7347	96	1-24	159.36	98.81
HCC1470_11	IRSEP	CAP AMK KAN OFX MOX ETH CS PAS	5	2	20	LAM1	412	295	707	251	958	1.3966	0.5830	0.7380	99	1-24	96.90	98.76
HCC759_09	IR	ETH	2	1	1	Beijing	623	414	1037	418	1455	1.5048	0.6102	0.7127	160	1-28	70.73	99.39
HCC916_10	IRSEP	CAP AMK KAN OFX ETH	5	2	1106	LAM4	373	281	654	238	892	1.3274	0.6044	0.7332	103	1-24	145.85	98.82
HPV105_09	S	nd	5	2	1752	LAM1	400	281	681	244	925	1.4235	0.6214	0.7362	37	1-22	591.50	98.93
HPV113_08	IRSEP	ETH	6a	3	54	MANU2	222	126	348	140	488	1.7619	0.5945	0.7131	62	1-24	167.28	99.94
HPV115_08	IRSEP	CAP AMK KAN OFX ETH	5	2	1106	LAM4	312	225	537	196	733	1.3867	0.7318	0.7326	76	1-21	179.43	98.82
HPV157_06	IS	nd	5	2	17	LAM2	397	287	684	248	932	1.3833	0.5760	0.7339	90	1-24	96.25	98.87
HPV50_09	Susceptible	nd	5	2	20	LAM1	397	291	688	238	926	1.3643	0.6554	0.7430	69	1-24	281.12	98.94
HPV51_09	Susceptible	nd	3c	2	137	X2	420	309	729	276	1005	1.3592	0.5677	0.7254	29	1-18	632.88	99.60
HPV65_08	Susceptible	nd	6a	3	Unknown	Unknown	247	160	407	162	569	1.5438	0.5837	0.7153	15	1-18	1149.11	99.57
HPV70_09	Susceptible	nd	5	2	1803	LAM1	380	277	657	224	881	1.3718	0.6343	0.7457	26	1-45	1410.21	99.65
HVNG1	IRSEP	CAP AMK KAN	5	2	20	LAM1	308	225	533	201	734	1.3689	0.7082	0.7262	76	1-24	154.30	98.78
IHMT134_09	IRSP	RFB ETH	5	2	20	LAM1	332	228	560	203	763	1.4561	0.6935	0.7339	83	1-24	171.49	98.77
IHMT149_09	IRSEP	RFB CAP AMK OFX MOX ETH	5	2	1106	LAM4	313	226	539	193	732	1.3850	0.6849	0.7363	75	1-21	168.54	98.81
IHMT194_11	IRSEP	RFB CAP AMK ETH	5	2	1106	LAM4	382	274	656	236	892	1.3942	0.6318	0.7354	83	1-24	47.61	98.96
IHMT288_95	IRSP	RFB ETH	5	2	20	LAM1	415	304	719	247	966	1.3651	0.6230	0.7443	98	1-24	197.48	98.84
IHMT295_08	IRSEP	RFB ETH	2	1	1	Beijing	528	337	865	358	1223	1.5668	0.7284	0.7073	127	1-59	181.48	99.18
IHMT308_08	IRP	RFB ETH	5	2	1106	LAM4	334	228	562	201	763	1.4649	0.7524	0.7366	76	1-21	205.47	98.79
IHMT359_03	R	nd	5	2	Orphan	LAM1	406	289	695	247	942	1.4048	0.6276	0.7378	31	1-22	727.91	98.93
IHMT361_08	IREP	CAP ETH	5	2	1106	LAM4	305	232	537	193	730	1.3147	0.6278	0.7356	70	1-24	191.20	98.80
IHMT69_11	IRSEP	RFB CAP AMK ETH	2	1	1	Beijing	609	420	1029	415	1444	1.4500	0.6307	0.7126	169	1-39	202.92	99.36
IHMT80_11	IRSEP	RFB CAP AMK ETH	5	2	1106	LAM4	380	277	657	236	893	1.3718	0.6318	0.7357	87	1-24	63.98	98.95
IHMT82_09	IRS	RFB CAP ETH	5	2	20	LAM1	313	221	534	190	724	1.4163	0.6808	0.7376	81	1-24	121.47	98.69

^a^First-Line: *I* - Isoniazid, *R* - Rifampicin, *S* - Streptomycin, *E* - Ethambutol, *P* - Pyrazinamide; Second-Line: *ETH* - Ethionamide, *KAN* - Kanamycin, *AMK* - Amikacin, *OFX* - Ofloxacin, *MOX* - Moxifloxacin, *RFB* - Rifabutin, *PAS* - Para-amino salicylic acid, *CS* - Cycloserine.

^b^Spoligotype inferred from SpolPred software (Coll et al., [89]).

^c^Small INDELs called by SAMtools from mapping to *M. tuberculosis* H37Rv.

^d^Relative to *M. tuberculosis* H37Rv.

*na*, Not available.

*nd*, Not done.

### Genotypic analysis

**Figure 1:**
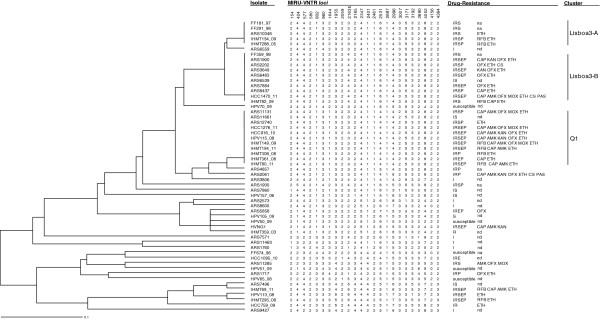
**MIRU-VNTR genotypic analysis of the 56** ***M. tuberculosis*** **isolates.** MIRU-VNTR dendrogram of the 56 *M. tuberculosis* clinical isolates subjected to WGS. First-line drug susceptibility testing: I, INH; R, RIF; S, STP; E, EMB; P, PZA. Second-line drug susceptibility testing: KAN, kanamycin; AMK, amikacin; CAP, capreomycin; OFX, ofloxacin; MOX, moxifloxacin; ETH, ethionamide; PAS, para-amino salicylic acid; CS, cycloserine; na, not available, nd, not determined.

The 24-*loci* MIRU-VNTR genotyping technique grouped the MDR-TB isolates into three major clusters: Lisboa3-A, Lisboa3-B and Q1 (Figure 1). Use of the 12-*loci* set groups Lisboa3-A and -B in a single cluster (Lisboa3, data not shown). Only the Lisboa3-B and Q1 clusters were found to be associated with XDR-TB isolates. Eight of the ten XDR-TB isolates belonged to either Lisboa3-B or Q1 cluster, and one of remaining two strains was found to be Q1-related, raising the possibility of ancestral Q1 XDR followed by posterior divergence from this cluster. No XDR-TB isolate was found to belong to Lisboa3-A cluster.

### Genomic analysis

All 56 clinical isolates underwent whole genome sequencing (WGS) The total number of identified SNPs (point mutations differing from H37Rv) ranged between 488–1465 (mean: 928.0, 26.7% in non-coding regions) (Table 1). Of the SNPs on coding regions, 58.5% were considered non-synonymous substitutions yielding a mean non-synonymous/synonymous ratio (N_s_/S) of 1.41 (Table 1). AG, CT, GA and TC transitions were found to be the most frequent substitution types (see Additional file 1), which is reflected by a mean transversion/transition ratio (T_v_/T_s_) of 0.62. Overall, across the 56 clinical isolates and 19 publicly available reference strains (F11, CDC1551, KZN1435, KZN4207, KZN605, KZN_R506, KZN_V2475, UT205, RGTB327, RGTB423, CCDC5180, CCDC5079, CTRI-2, BTB05_552, BTB05_559, S96_129, HN878, R1207, and X122), 9419 genome-wide SNPs were identified by mapping to the reference genome of *M. tuberculosis* H37Rv. The number of small insertions and deletions (indels) detected upon read mapping ranged between 15–175 indels per isolate with a size between 1–59 bp (Table 1).

### Global phylogenetic analysis using WGS

Using WGS data, the 56 clinical isolates and 19 publicly available strains were assigned into established six SNP Clusters Groups (SCG) and three Principal Genetic Groups (PCG) [14, 20]. Overall, at least one isolate belonging to each SCG and subgroups was included in the subsequent analysis. Forty-four (78.6%) of the 56 clinical isolates belonged to SCG 5, reflecting the high prevalence of these strains in Lisbon Health Region (Table 1).

**Figure 2:**
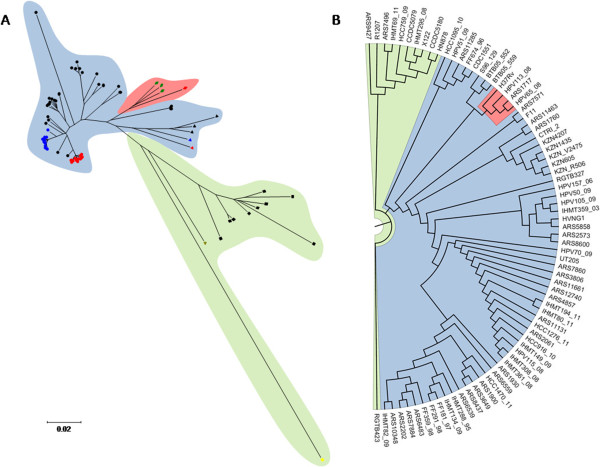
***M. tuberculosis*** **genome-wide SNP-based phylogeny.** Phylogenetic tree **(A)** and cladogram **(B)** of the initial 56 clinical isolates plus 20 *M. tuberculosis* public genomes. PGGs are highlighted in green (PGG1), blue (PGG2) and red (PGG3). A - Isolate-depicting symbols are representative of the different SCGs found in the tree: SCG 1 (yellow square), SCG 2 (black squares), SCG 3a (green triangle), SCG 3b (red triangle), SCG 3c (blue triangle), SCG 4 (black triangles), SCG 5 (circles), SCG 6a (green diamonds), SCG 6b (red diamond). Lisboa3 and Q1 strains are represented by red and blue circles (within SCG 5), respectively.

A phylogenetic tree was inferred from a set of 9419 genome-wide SNPs (Figure 2). It reveals that the two main genetic clusters associated with XDR-TB in the region, Q1 and Lisboa3, constitute two genetically close but distinct clades within the SCG 5. The MIRU-VNTR Lisboa3-A cluster was found to form a monophyletic group within the Lisboa3 clade. The MIRU-VNTR Lisboa3-B clade designation was therefore considered as paraphyletic in the light of a genome-wide SNP phylogeny. The sequenced strain closest to the Lisboa3-Q1 clade is *M. tuberculosis* UT205, a virulent Colombian isolate that according with the present phylogeny shares a more recent common ancestor with Q1 strains than these do with Lisboa3 strains.

### Global evolution through large sequence polymorphisms

Genomes in the *M. tuberculosis* complex can downsize, through Large Sequence Polymorphisms (LSP) or Regions of Difference (RD), and 89 have been previously identified [21–23]. Across the 75 isolates, 29 (of 89) were detected as absent in at least one isolate (see Additional file 2). The most prevalent RDs detected were RD149 (64 isolates), RD152 (45), RD174 (43), RD3 (64), RD6 (54) and RD^RIO^ (43). As expected, all 43 strains bearing the RD174 (LAM) deletion also had the RD^RIO^ deletion [24]. Both deletions constitute a distinct sub-lineage within the Euro-American lineage [23] and were detected only among SCG 5 strains. UT205, like the Q1 and Lisboa 3 samples, had both deletions, confirming its phylogenetic proximity with these M/XDR associated strains (see Additional file 2).

All nine isolates from the SCG 2 had the RD105 deletion characteristic of the East-Asian clade. Of these, other RD deletions were present (RD207 9 isolates, RD181 8, RD142 2). Moreover, other RD deletions associated with specific lineages were detected: RD750 (East-African-Indian lineage, SCG 3a, 1 isolate), RD115 (Euro-American lineage, Americas-Europe sublineage, SCG 5, 8), RD183 (Euro-American lineage, Americas-Europe sublineage, SCG 3c, 1) RD193 (Euro-American lineage, Americas-Europe sublineage, SCG 4, 3), RD219 (Euro-American lineage, Americas-Europe sublineage, SCG 6a, 3), RD761 (Euro-American lineage, South Africa sublineage*,* SCG 5, 1 (F11 strain)) and RD724 (Euro-American lineage, Central Africa sublineage, SCG 5, 3).

No RD region was found to be absent in RGTB423 and only RD^RIO^ deletion was detected in RGTB327. Strain RGTB423 has been found to belong to SCG 1 and PGG 1 [25], but in-silico PCR analysis showed that the strain had the *pks15/1* 7 bp frameshift deletion and the TbD1 deletion indicative of a modern Euro-American strain [23]. Nevertheless, this classification is incongruent with the SCG and PGG classification [11]. On the other hand, RGTB327 was found to have the RD^RIO^ deletion only and *in silico* PCR of the *pks15/1* and TbD1 loci also pointed towards a modern Euro-American strain, despite the fact that deletion RD174 was not detected. Further sequencing of these two assembled strains may be required to resolve incongruences.

**Figure 3:**
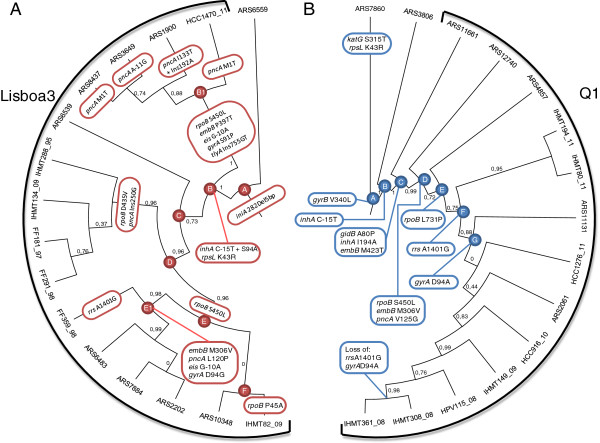
**Microevolution from susceptible TB towards MDR- and XDR-TB.** Lisboa3 **(A)** and Q1 **(B)** subtree cladograms highlighting the microevolutionary path towards MDR and XDR within these two phylogenetic clades. Mutations acquired in genes associated with first and second-line drug resistance are shown in branch or associated node.

Other structural variants were also searched employing different methodologies (see Additional files 3, 4, 5, 6, 7 and 8). From the deletions described in the supplementary material we highlight characteristic deletions for the Lisboa3 clade and the ARS6559 isolate (complete Lisboa3 subtree in Figure 3) (112 bp, position 2727803, PPE41 gene), as well as the Q1 clade strains (297 bp, position 3929891, ORF PE_PGRS53). Both deletions were also validated by mapping coverage, but further laboratory confirmation is required.

### Microevolution towards multidrug and extensively drug resistance

Given the relative high number of sequenced strains present in both Lisboa3 and Q1 clades it was possible to trace the microevolutionary path reflecting the genomic changes accompanying the resistance acquisition process. We considered the subtrees containing the Lisboa3 and Q1 clades plus one or two strains for the Lisboa3 and Q1 subtrees, respectively, included as outgroups for the ensuing analysis (Figure 3). In particular, we inferred the changes in candidate resistant gene mutations at the nodes of the trees.

**Figure 4:**
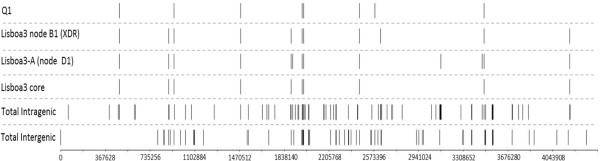
**Mapping of IS** ***6110*** **insertion sites.** Genomic distribution of total mapped IS*6110*, intra and intergenic, and insertion sites found among Lisboa3 and Q1 isolates. Lisboa3 core and Q1 lanes depicts all insertion sites that are common to all Lisboa3 and Q1 clade isolates, respectively. Lisboa3 node B1 comprises a XDR-TB lineage shown here with an extra IS6110 copy. Lisboa3-A (node D1) are shown here to bear three additional IS6110 copies when compared with the Lisboa3 core.

The Lisboa3 subtree, including the outgroup strain ARS6559, was found to be characterized by a 5 bp deletion on the *iniA* gene. There is a common acquisition of high-level INH resistance through a *inhA* double mutation (in node B). The data also reflect the acquisition of RIF resistance in three separate occasions, twice in the Lisboa3-B strains by a *rpoB* S450L (equivalent to *E. coli* S531L) and in Lisboa3-A lineage by a *rpoB* D435V (equivalent in *E. coli* to D516V). Acquisition of XDR can be seen in the two branches: the first by acquisition of an *eis* G-10A, *gyrA* S91P and tlyA Ins755GT mutations (node B1); and, by an *eis* G-10A and *gyrA* D94G mutations (node E1). EMB resistance is likely to have been acquired twice by *embB* M306V and P397T mutations. The latter mutation has been previously reported in one EMB resistant isolate [26]. PZA resistance was found to be acquired on multiple independent occasions through *pncA* mutations.

The Q1 subtree included two other Q1-related strains as outgroups. Here, it is possible to distinguish the acquisition of INH low-level resistance by an *inhA* C-15 T mutation (node B) from the acquisition of a higher INH resistance level by an *inhA* missense mutation (I194A, node C) [27]. Some of the isolates present in the subtree were found outside the Q1 MIRU-VNTR cluster, but share more recent common ancestors with other strains in the clade, potentially indicating subsequent MIRU-VNTR divergence. The Q1 clade has, therefore, been defined as all isolates bearing the *gidB* A80P mutation characteristic of this cluster and associated with STP intermediate-level resistance previously described by some of us [10]. A more linear resistance acquisition dynamic was found for this clade. EMB resistance was acquired on two possible occasions, through an *embB* M423T (node C) and M306V (node D) mutations. RIF resistance development, leading to MDR-TB, was found to be acquired by a *rpoB* S450L mutation (node D), although a second mutation on *rpoB* (L731P) was later developed (node E). Resistance to PZA, injectable second-line drugs and FQs occurred once by mutations on *pncA* (V125G, node D), *rrs* (A1401G, node F) and *gyrA* (D94A, node G), respectively. Interestingly, isolates IHMT308_08 and IHMT361_08 did not show the two latter mutations in *rrs* and *gyrA* genes, and therefore inconsistent with both strains positioning in the Q1 subtree.

A further observation is that M/XDR development in the Lisboa3 subtree appeared to be accompanied by a higher genomic diversification, translated in the number of SNPs and small indels (Additional files 9 and 10). This observation is probably in line with an earlier emergence of the Lisboa3 clade and prolonged circulation in the community leading to a higher intra-clade diversity when compared to Q1 strains. Moreover, isolates from the Lisboa3 and Q1 clades were found to bear a mean proportion of 0.73% (range: 0.2-1.8%) and 0.85% (range: 0.2-1.6%) unique SNPs, respectively, in comparison with the total SNP count of each strain. Both clades were found to share a pool of 654 (67.7-90.3%) and 626 (68.2-85.2%) common SNPs, respectively (Additional file 11). This intra-cluster degree of genomic uniqueness is comparable with the data reported by Niemann *et al.* for the comparison of two Beijing isolates from the same outbreak clone [13].

### Mutational compensation for RIF-resistance

The acquisition of compensatory mutations following resistance development has been proposed as a possible mechanism to reduce the fitness cost carried by drug resistance [28]. More recently, *rpoA* and *rpoC* genes were found to harbor putative RIF resistance compensatory mutations [18, 29, 30]. The microevolutionary analysis of Lisboa3 and Q1 clades led to the identification of two possible compensatory mutations in *rpoC* (K1152Q, node B to B1 in the Lisboa3 subtree; see Additional file 12) and *rpoB* (L731P, node D to E in the Q1 subtree; see Additional file 13) leading to RIF resistance acquisition. The *rpoA* and *rpoC* genes were screened for mutations in all isolates. On the overall 13 different non-synonymous mutations were found, of which only 6 occurred among MDR/RIF-resistance isolates (Table 2). The impact on protein function was inferred by computation of SIFT scores [31]. Only three mutations occurring in *rpoC* (see Additional file 14) were predicted to affect protein function with SIFT scores equal to 0.00, resulting from the comparison of 189 sequences represented at each position (Table 2). The remaining mutations were predicted to be tolerated and yielded higher SIFT scores (>0.05), resulting from the comparison of 171–189 sequences representing each position tested (Table 2).

**Table 2:** **Candidate RIF resistance compensatory mutations found in RpoA, RpoB, and RpoC among RIF-resistant isolates with other RIF resistant associated mutations in RpoB**

Protein	Mutation	SCG	No. of Isolates	SIFT Score
RpoA	E184D	2	1	0.09
RpoB	P45A	5	1	0.01
RpoB	T328N	2	1	0.03
RpoB	L452P	5	2	0.00
RpoB	V496A	6a	1	0.10
RpoB	D634G	5	1	0.49
RpoB	L731P	2, 5	13	0.00
RpoB	E812G	2	1	0.08
RpoB	I1106T	5	2	0.00
RpoC	G442C	5	1	0.00
RpoC	W484G	2	1	0.00
RpoC	D747G	4	1	0.35
RpoC	K1152Q	5	4	0.00
RpoC	S1287L	6a	1	0.23

We also screened the remaining RNA polymerase subunits, RpoB and RpoZ, but only eight non-synonymous mutations were identified in RpoB, concomitantly with other RIF resistance associated mutations in RpoB (Table 2). Five RpoB mutations (P45A, T328N, L452P, L731P and I1106T) were predicted to affect protein function after SIFT score analysis (SIFT score <0.05) (Table 2).

### Insertion sequence (IS) mapping and functional consequences for genomic stability

Transposition events from ISs can have a profound effect on strain physiology given the possibility of interference with gene expression by ORF knock-out or gene upregulation resulting from upstream transposition [32, 33]. For all strains included in the phylogenetic analysis, we attempted to map the site of all ISs annotated as mobile elements in the genome of *M. tuberculosis* H37Rv, namely IS6*110*. Some complex inversions were found to be predominantly transpositional events from multi-copy mobile-elements, such as IS*6110*. The analysis revealed the presence of IS*6110*, IS*1081*, IS*1547*, IS*1557* and IS*1558* in multiple copies, but differing in size or annotated sequence at both extremities. For this reason these ISs have been excluded from the mapping analysis.

Variability was only observed for IS*1561* and IS*1532* (Additional file 15). As expected, IS*1561* was not detected in all isolates bearing the RD^RIO^ deletion, whereas IS*1532* is absent in isolates bearing the RD6 deletion found on different SCGs. For IS*6110*, a total of 251 candidate insertion sites have been obtained (Additional file 16), classified as of high (160), medium (18) or lesser (73) confidence. Almost half (125 (49.8%)) of the 251 ISs were observed on the positive strand. A total of 105 (41.8%) insertion sites were found to be intergenic, from which 64 (25.5%) were in the same orientation with an upstream ORF, known to exert a putative upregulatory effect. For these latter insertion sites the distance from the 3′ end to the upstream ORF ranged between 0–939 bp (47 (18.7%) less than 300 bp). Thirty-three sites were found to be within PE/PPE genes, while three other insertion sites were located 18–38 bp upstream of a PPE gene.

Lisboa3 and Q1 clades were found to share 7 IS*6110* sites but were differentiated by IS*6110* insertions on positions 889015 (intergenic) and 4183431 (Rv3732 knock-out) for Lisboa3 and, on 2582457 (intergenic) for Q1 isolates (Figure 4). Moreover, we have found that strains belonging to Lisboa3-A MIRU-VNTR cluster (*rpoB* D435V clade on Figure 3-A) share three distinct IS6110 insertion sites on Rv1682 (position 1906425), Rv2818c (position 3125900) and Rv3096 (position 3465467). Strains from the XDR-TB Lisboa3 B1 clade (Figure 3-A) share a distinctive IS*6110* site on the *plcC* gene (position 2628462). Although no common IS*6110* site was found for the SCG 5 strains, SCG 2 strains were found to share three IS*6110* sites: an intergenic site on position 888786; on Rv1754c (position 1986639); and, on Rv2820c (position 3127931). SCG 4 strains were found to also share three IS6110 sites on *mmpS1* (position 483580), PPE46 (position 3377326) and PPE47 (position 3379768). One hundred and fifty-three (60.0%) sites were found to be specific to a single isolate.

Interestingly, an IS*6110* insertion in the NTF locus (position 3493907) was detected in six out of the eight Beijing strains included in the analysis, which is a characteristic of the Beijing/W family [34, 35] (Additional file 16). Hence, two of the three Beijing isolates recovered in Lisbon Health Region were found to belong to the Beijing/W family. No relation with the New York City Beijing/W MDR clade was found as a second insertion in the NTF locus was not detected in any strains [34, 35]. Curiously, a SCG 6a strain (HPV113_08) shared the latter insertion site with the Beijing/W strains, although only one end was detected which can be indicative of another genomic rearrangement. A SCG5 strain (HPV157_06) was found to have an IS*6110* 67 bp upstream of the characteristic IS*6110* insertion site of the Beijing/W family, however in a different orientation. Both insertion sites are found within Rv3128c. This latter gene has an in-frame amber nonsense mutation in H37Rv and for this reason any functional consequence of IS*6110*-mediated ORF disruption is highly questionable.

Strains belonging to PGG2 were found to have a significantly lower number of IS*6110* copies when compared with PGG1 strains (Kruskal-Wallis test, p <0.001). Given the reduced number of PGG3 strains no statistical comparison was possible to perform.

### Differential substitution ratios highlight different genomic adaptation strategies

A statistically significant difference in the N_s_/S ratio was observed between Lisboa3/Q1 and Beijing strains and others, but only the Lisboa3 and Q1 result met a multiple comparison threshold (Additional file 17). The only significant T_v_/T_s_ ratio difference occurred for differences between Lisboa3 and Q1 clusters (Q1 greater, mean difference: 0.045, p =0.033) (Additional file 17).

These ratios were also found to vary across the genome and across the different Clusters of Orthologous gene Groups (COGs). For each strain, we have computed the N_s_/S and T_v_/T_s_ ratio for the different genomic quadrants and for each COG. Overall quadrant N_s_/S and T_v_/T_s_ comparison, showed that N_s_/S ratio varied along the chromosome such that the second quadrant had a lower N_s_/S ratio when compared with the other three quadrants and that the first quadrant had the highest N_s_/S mean ratio (Kruskal-Wallis, p <0.001) (Additional file 18). No statistical difference was observed between the third and fourth quadrant. Regarding the T_v_/T_s_ ratio, an approximately inverse situation was found as no statistical difference was observed between the first, third and fourth quadrants. The second quadrant showed however, a significantly higher T_v_/T_s_ ratio than the three other quadrants (Kruskal-Wallis, p <0.001) (Additional file 18).

When these results were stratified by genetic clade, it was found that in the first quadrant the Beijing strains showed a statistically lower N_s_/S ratio upon comparison with Q1 and other non-clustered (NC) strains, but not Lisboa3 (Additional file 19). No statistical difference was found in this quadrant for T_v_/T_s_ ratio. In the second quadrant, Lisboa3 strains showed a statistically significant reduced N_s_/S ratio compared with the other three groups of strains, while Beijing strains presented a higher N_s_/S ratio than the remaining groups (Additional file 19). Inversely, the T_v_/T_s_ ratio on the second quadrant was significantly higher for Beijing strains when compared to Q1 and other NC strains, but not to Lisboa3 strains (Additional file 19). The analysis of the third quadrant showed no statistical difference for Ns/S ratio while Beijing strains showed a higher T_v_/T_s_ ratio on comparison with Lisboa3 and other NC strains, but not Q1 strains. Lisboa3 strains showed a reduced T_v_/T_s_ ratio on this latter quadrant when compared to all other groups. In the fourth quadrant, only a statistical difference was observed for a Q1 reduced N_s_/S ratio when comparing with the other strain groups and no significant difference was observed for the T_v_/T_s_ ratio (Additional file 19).

These results show that the N_s_/S and T_v_/T_s_ ratio measures appear to vary on a strain and chromosome region dependent mode. Data stratification by isolate and quadrant showed that the T_v_/T_s_ ratio was found to correlate negatively with the N_s_/S ratio (Pearson, p <0.001). Correlation between overall isolate N_s_/S and T_v_/T_s_ ratio was also attempted but no correlation was found (Pearson, p =0.433).

The comparison of the N_s_/S and T_v_/T_s_ ratios across the different COGs also yielded strain dependent results. On comparison with the other three strain groups: Lisboa3 strains showed higher Ns/S ratios on COG groups D (Cell Cycle Control, Mitosis and Meiosis) and P (Inorganic Ion Transport); Q1 strains showed higher Ns/S ratios on COG group V (Defense Mechanisms); and, Beijing strains showed higher N_s_/S ratios on COG groups F (Nucleotide Transport and Metabolism), K (Transcription), N (Cell Motility), O (Post translation Modification, Protein turnover and Chaperones) and Q (Secondary Metabolites Biosynthesis, Transport and Catabolism) (Additional file 20). Regarding the T_v_/T_s_ ratio no significant difference was observed for Lisboa3 strains, but higher ratios were observed for Q1 strains in COG groups J (Translation), L (Replication, Recombination and Repair), M (Cell Wall, Membrane Biogenesis) and, for Beijing strains in COG group C (Energy Production and Conversion) (Additional file 21).

These results support the notion of a differential mode of evolution and adaptation to the human host by accumulation/selection of a higher degree of non-synonymous mutations at genes belonging to specific functional categories.

According to recent work by Namouchi et al. [36], the N_s_/S ratio varied along the phylogenetic tree, such that terminal branches had a higher N_s_/S ratio than inner branches. We have computed the N_s_/S and T_v_/T_s_ ratio for the inner nodes assigned in the subtrees in Figure 4 and compared with the respective ratios calculated for the tips of the subtrees. Contrary to the data of Namouchi et al. [36] we have verified that both subtrees had ≈ 6% and ≈ 12% lower Ns/S ratios at the tips of Lisboa3 and Q1 subtrees, respectively, when compared with the inner nodes of the tree (Independent t-test, p <0.001). For the T_v_/T_s_ ratio, the opposite was found: higher T_v_/T_s_ ratios were observed at the tips in comparison with the inner nodes (Mann–Whitney test, p <0.001).

## Discussion

### *M. tuberculosis*genomic distinctiveness in Lisbon

For at least two decades the Lisbon Health Region in Portugal has been characterized by a high-level of drug resistance, at first MDR-TB, and later XDR-TB, mainly caused by a particular group of strains: the Lisboa family. Presently, this drug resistance is due almost in its entirety to an endemic circulation of the Q1 and Lisboa3 phylogenetic clades. Present data from 24-*loci* (not 12-*loci*) MIRU-VNTR allowed the subdivision of the Lisbon3 cluster in two other clusters herein designated as Lisboa3-A and –B. This data suggests two independent outbreaks, over the years, dated back to the 90s when the discrimination of Lisboa strains was identified by distinct *rpoB* mutations [8]. The Q1 spoligotyping data has revealed that this cluster is in fact intimately related with the B cluster identified in the 90s outbreak (unpublished data). Phylogenetic analysis based on previously published sets of SNPs [14, 37] revealed that Lisboa3 and Q1 strains formed distinct monophyletic evolutionary clades within the SCG 5 and PGG 2. Interestingly, *M. tuberculosis* F11 and the XDR-TB associated KZN strains, both originating from South Africa, also belong to SCG 5. Nevertheless a clear distinction is highlighted in the proposed phylogeny. This distinctiveness is also reflected by the RD comparison, but Lisboa3, Q1 and KZN strains appear to have an incongruent phylogeographic association using the RD typing. All these strains belong to the Euro-American lineage according to the RD classification proposed by Gagneux et al. [23]. However, the KZN strains included in the analysis showed to be positive for RD115, associated with an Americas/Europe sublineage, despite the fact that these strains are a major public health concern in South Africa, namely, the XDR-TB outbreak in KwaZulu Natal [38, 39]. The Lisboa3 and Q1 strains were on the other hand positive for RD174, associated with a West-African sublineage, but constitute a major public health concern in Europe. Present knowledge recognizes that RD174 is also associated with RD^RIO^, an LSP that has initially been discovered in Rio de Janeiro, Brazil but was later found to be widespread. Historic ties connect Portugal, Brazil and West African Countries and a possible ancestor for these two clades might lie in Africa, more specifically on Portuguese Speaking African Countries. These phylogeographic incongruences are consistent with human migratory events out from, and back into, the African continent [12]. Moreover, these results also highlight that more is still needed to fully grasp the genetic diversity present within the SCG5 and LAM family as it encloses a high genetic diversity allied with a broad geographical distribution [40].

Another question still seems pertinent as to which selective advantages do these two clades possess allowing such high prevalence in this setting especially since other strains, e.g. pre-XDR-TB Beijing strains, also do circulate but at an apparent lesser prevalence? TB caused by RD^RIO^ strains has shown to be associated with weight loss, hemoptysis, higher bacillary loads and progression to cavitary disease [21, 41]. This deletion encompasses several PPE genes that have shown to be a potential source of immune variation (reviewed in [42, 43]) and hence, may constitute a pathogenic adaptation strategy to immune evasion. Higher bacillary loads are associated with a higher secondary case rate [44–46] and if in fact the absence of these genes truly plays an important role towards an increased virulence, or even transmissibility, it may be a factor that has contributed to the high prevalence of RD^RIO^ strains in this setting simultaneously contributing to the emergence and spread of M/XDR-TB strains.

Besides previously described RDs, the additional structural variants that were identified and that may be clade-specific could carry functional consequences that reflect host adaptation and selection. The finding that a 112 bp deletion is present among Lisboa3 clade strains, with a more restricted distribution than RD^RIO^, affecting gene PPE41 might also provide additional clues and contribute to a higher virulence or transmissibility. PPE41 has been previously described has having an immunodominant nature and shown to activate a CD4^+^ and CD8^+^ mediated T cell response leading to an enhanced IFN-γ response as well as induce a strong humoral response [47, 48]. The deletion found might constitute a means of immune evasion and constitute a selective advantage over other circulating strains. More specifically, a stronger humoral response to PPE41 was found among extra-pulmonary TB patients [48]. The selective advantage provided by this deletion might therefore also be related with the fact that Lisboa strains were first identified among HIV-infected patients, which is associated with an increase in extra-pulmonary TB.

### Phylogenetic context and microevolutionary trajectory of Lisboa3 and Q1 clades

The use of SNPs as molecular markers has contributed to an improved understanding of the evolutionary history of the *M. tuberculosis* complex. In the present study, given the availability of genomewide SNP data, a SNP-based phylogeny was deduced from the genomic data and, overall, the proposed phylogeny appears to be consistent with other SNP-based phylogenies although as already pointed out: SCG 3 does not exist as a monophyletic lineage but instead as a paraphyletic one. The original report by Filliol *et al.*[20] proposed a minimum number of sixteen SNPs that allowed assignment of any strain to an SCG but not to its subgroupings. A later erratum showed that SCG 3a belonged in fact to PGG1 while SCG 3b and 3c belonged to PGG2 as confirmed by our results. Alland et al. [37] proposed instead a set of nine SNPs that allowed strain assignment to any SCG and each subgroup [37].

The phylogeny constructed in the present study contributes nevertheless to demonstrate the uniqueness of Lisboa3 and Q1 strains in a global context and will comprise a future framework for genome-wide association studies (GWAS).

The phylogeny proposed also enabled a microevolutionary perspective on the path towards MDR and XDR. As expected, in the Lisboa3 and Q1 clades, INH resistance was found to be mediated by double *inhA* promoter/structural mutations, recently described by some of us to contribute to INH high-level resistance [27]. The acquisition of *inhA* C-15 T mutation was found to have occurred independently in both lineages, and in Q1 cluster it was possible to determine that C-15 T mutation was acquired at a first stage of INH high-level resistance development. In Lisboa3 it was not possible to determine which mutation appeared in the first place since no Lisboa3 isolate with a single *inhA* mutation was found. Recent work by Fenner et al. [49] suggested that *inhA* promoter mutations, more specifically C-15 T mutation, might be associated with Lineage 1 (Indo-Oceanic/SCG 1) [11, 49]. Nevertheless, another earlier study from Brimacombe et al. [50] showed that SCG 1 and 5 had all the mutations of interest towards INH resistance [50]. In our view, the fact that INH resistance in both Lisboa3 and Q1 clades is associated with *inhA* mutations, instead of the of the more usual KatG mutations, is possibly related with selective pressures exerted by the drug regimen itself.

The analysis of Lisboa3 subtree has further highlighted the M/XDR evolutive process in this clade. We have recently proposed an evolutionary path regarding drug resistance acquisition dynamics based on the acquisition of an *eis* promoter mutation as the first-step from MDR to XDR [6]. However the SNP phylogeny proposed is consistent with a twice and independent acquisition of an *eis* promoter mutation. Given this phylogeny it is not possible to establish any order of mutation acquisition. Nonetheless, instead of a single event, our analysis supports multiple development of XDR-TB in the same phylogenetic clade. Two different transmission chains involving strains with the RpoB S450L, instead of one, are also more likely since it is proposed that this mutation has also been acquired twice and independently [8].

Also important, he Lisboa3 XDR lineage characterized by *gyrA* D94G and *eis* G-10A mutations (node E1) will most likely present resistance to KAN, but not to CAP and AMK. If drug susceptibility testing to KAN is not included in the standard second-line drug panel of tested drugs, the strains belonging to this lineage will have an undetected XDR phenotype. An exception to this is the strain FF359_98 that bears a *rrs* A1401G mutation that leads to high-level KAN, AMK and CAP resistance [51].

One striking phylogenetic incongruence was found in two Q1 strains that lacked both second-line injectable drug and FQ genetic resistance determinants and at the same time sharing a recent common ancestor resistant to these two classes of drugs. These two strains were genotypically and phenotypically susceptible to amikacin (AMK), capreomycin (CAP) and any of the FQs tested. Two explanations may be considered: a phylogenetic misplacement, although the branches had a good statistical support or, these strains may descend from a reverter ancestor. Although theoretically possible, events such as these may be extremely rare. Only one report has documented an in-patient reversion of an isogenic strain from INH resistant to susceptible [52].

### Compensatory evolution and RIF-resistance

The acquisition of further mutations in *rpoA*, *rpoB* or *rpoC* genes following RIF resistance development was recently demonstrated, using *Salmonella* as a model organism, to have an important role in fitness compensation, leading to a reduction in the doubling-time to values closer to the wild-type [53].It as also been demonstrated that *rpoC* gene has been target of convergent evolution [54]. In our microevolutionary analysis we have detected a RpoC mutation (K1152Q) occurring in the same branch as a RpoB S450L (equivalent to S531L in RpoB *E. coli* numbering). It is the first description of a putative compensatory mutation within the Lisboa3 clade, contributing to the success of one of its sub-lineages through the amelioration of the resistance fitness cost [28]. RIF compensatory evolution has been the subject of two recent studies that showed a high prevalence of *rpoA* and *rpoC* mutations mapped to the RpoA-RpoC interaction region [29, 30, 55]. The *rpoC* mutation described in a Lisboa3 sub-lineage does not fall in this region, nor was it described in these studies [29, 30]. Nevertheless, two other putative compensatory mutations mapping to the RpoA-RpoC interaction region were found in other isolates not belonging to the Lisboa3 or Q1 clades (Additional file 14). The putative role of these two latter mutations is only substantiated by the bioinformatic analysis of residue conservation. However, the putative compensatory role of the Lisboa3 K1152Q RpoC is further substantiated by their co-occurrence in the same branch as the RIF resistance determining mutation in *rpoB*. Furthermore, none of these putative compensatory mutations was previously described and may constitute novel polymorphisms associated with molecular RIF resistance compensation [18, 29, 30].

RpoB mutational analysis also allowed the identification of five putative compensatory mutations, of which one (L731P) was found to be acquired in the Q1 clade following RIF resistance acquisition through another *rpoB* mutation. This latter mutation was found to be homoplasic as it was also detected in different SCGs, which also points towards the usefulness of this mutation to counteract fitness costs imposed by the acquisition of other RIF resistance associated mutations. Mutations outside the RIF resistance determining region on *rpoB* gene have been described previously on RIF-resistant isolates with no mutations on this region [56, 57]. The mutations herein described as putatively compensatory were only considered as such if a mutation in the RRDR was already present providing further support for the compensatory role of the former.

The role of compensatory mutations in other *loci* and associated with compensation to resistance to other drugs than RIF have been identified and studied, namely, mutations on *ahp*C for INH or on *rrs* for second-line drug aminoglycosides [58–60]. Nevertheless, no compensatory mutations were identified in these genes (data not shown).

Still, it is yet possible that other mechanisms underlying resistance or compensation might lie elsewhere in the genome as even the role that synonymous SNPs play in gene expression must be reckoned with. Such an example in *M. tuberculosis* comes from a recent and elegant study by Safi *et al.*[61] in which a synonymous SNP on Rv3792 was found to act as an hypermorphic mutation on a downstream gene (*embC*), leading to an increase in EMB resistance [61]. In the same study, another type of mutation was found to be a key player at the multistep process of EMB resistance development – a neomorphic mutation on gene Rv3806c that increased the turnover of the decaprenylphophoryl-β-D-arabinose pathway, which also led to an increase in EMB resistance [61]. Two other recent studies from Zhang *et al.*[62] and Farhat *et al.*[54] also point to other genes that may be at play and under positive selection concerning drug resistance in *M. tuberculosis*[54, 62]*.* It becomes clear that functional characterization of the significant portion of the *M. tuberculosis* genes of unknown function must catch up the pace of high-throughput sequencing if a broader understanding of the genomic adaptation process is to be obtained.

### IS*6110*transposition role in gene integrity and regulation

Insertion site mapping revealed a high genomic stability of insertion sequences other than IS*6110*. In fact, we have verified that only deletion events were responsible for variability regarding presence/absence of an insertion sequence other than IS*6110*. On the other hand, as demonstrated in this study IS*6110* is a highly polymorphic marker, probably due to its rapid transposition rate [63, 64].

The finding that 65.5% of the IS*6110* insertion sites mapped were located intragenically is in line with previous reports [65]. Considering that ≈ 91% of *M. tuberculosis* genome is composed by coding regions [66], it highlights the deleterious effects of transposition into certain genes essential to viability or to the successful completion of the pathogen’s infectious cycle. PGG1 strains, including the Beijing strains, were found to bear a higher number of IS*6110* copies than PGG2 strains. IS*6110* copy number is presumed to be under negative selection [67], however, in certain circumstances, it is the insertion site *per se* that might provide a selective advantage and not the copy number.

Considering the data obtained in this study, IS*6110* is unarguably the species’ most important mobile element when considering transposition impact on genomic integrity. IS*6110* appears to have an important role in genomic re-shaping towards adaptation either through localized disruption of putative antigenic targets (e.g. PPE/PE genes) or through its mobile promoter activity located in the IS*6110* 3′ end, capable of inducing transcription or upregulating the expression of downstream genes under stressful conditions [68]. We have found a considerable number of insertion sites to be within PPE genes and a more reduced number of sites to be upstream of PPE genes. PPE genes appear to have been positively selected in pathogenic mycobacteria, have important immune and antigenic potential, and some can induce a shift towards a Th2-type response [42, 43]. Not only the IS*6110*-mediated disruption of PPE genes might constitute a mean of immune evasion but it is also conceivable that upregulation of specific PPE genes might affect the Th1/Th2 response balance.

Remarkably, Lisboa3 and Q1 did not show any IS*6110*-mediated disruption of a PPE ORF, nor did we found any IS*6110* upstream of a PPE gene. This fact perhaps demonstrates a different mode of evolution and host adaptation that does not require PPE gene modulation through IS6110 transposition.

Nevertheless, the maximum distance between an IS*6110* and a downstream gene so that this 3′ promoter can exert its influence on gene expression is unknown. The results reported by Safi et al. [68] demonstrate that an IS*6110* in *M. tuberculosis* 210 located 297 bp upstream of Rv1468c was associated with a threefold increase in transcription upon macrophage infection. Our results show that 15% of the mapped sites are located upstream of an ORF in proper orientation and at a distance of less than 300 bp which, at the light of present knowledge, fulfills the necessary assumptions to exert a putative upregulatory effect on those ORFs. Also considering the diversity of ORFs interrupted by IS*6110* copies, gene knock out studies and assessment of downstream gene expression are necessary if a functional role for specific transposition events is to be established.

Spoligotyping lineage association with specific IS*6110* sites has already been demonstrated, highlighting the phylogenetic informativeness of this marker [69]. Our results also support an association of specific IS*6110* sites with strain lineage at both global and local levels.

### Genome-wide SNP dynamics

Notably, the comparison of the distribution of SNPs by COG showed that N_s_/S ratios vary through COG in a lineage-dependent manner. Although, Lisboa3 and Q1 isolates might be overrepresented in the analysis due to the high prevalence in the community, we have shown that Lisboa3 and Q1 present statistically different N_s_/S ratios from the remaining isolates. We propose that differences in N_s_/S COG might highlight different evolution strategies selected during host-pathogen interaction and adaptation.

Moreover, an overall higher N_s_/S ratio was observed for the first quadrant and an overall lower N_s_/S ratio was observed for the second quadrant revealing heterogeneous N_s_/S ratios negatively correlated with the T_v_/T_s_ ratio. The biochemical nature behind this T_v_/T_s_ ratio heterogeneity requires further studies as it may be driving localized higher non-synonymous mutation rates with functional impact on strain evolution. The precise genes affected by non-synonymous mutations within these COG categories merit further studies as each COG includes a considerable number of genes, that mutated might enhance the transmissibility or drug resistance, and should be analyzed in a systems biology perspective using *in silico* models [70–72].

The finding that terminal branches of the subtrees analyzed had lower N_s_/S ratios than the inner branches was contrary to the findings of Namouchi et al. [36]. Namouchi et al. suggested that non-synonymous changes might be purged by natural selection yielding higher N_s_/S ratios. However, an opposite view is also possible: non-synonymous mutations are favored by natural selection yielding the same higher N_s_/S ratio, especially as a mean of adaptation in an organism devoid, or with low, horizontal gene transfer such as *M. tuberculosis*[73, 74]. From our data, we can say that non-synonymous mutations may be favored in the inner branches of both subtrees as a mean to develop and adapt to drug resistance, yielding a higher N_s_/S ratio that is consistent with a reduced purifying selection [12]. Nevertheless, the differences between the results from both studies might lie in the fact that the N_s_/S ratio analysis herein presented was performed for two sets of strains that are in a much more closer time frame in order to understand microevolution within two clades.

### WGS and molecular epidemiology

In the present study we have shown that in Lisbon, Portugal, where the MDR-TB situation had already escalated to a XDR-TB situation, it is mainly caused by transmission of two unique phylogenetic clades. We have previously shown that XDR-TB was already a reality in Portugal during the 1990s [6] but noteworthy, the data from the present study clearly shows that these strains belong to the same phylogenetic Lisboa3 M/XDR sublineages that are still presently in circulation. The uniqueness of these strains was revealed by a distinct phylogenetic placement within SCG 5.

The Lisboa3 clade belongs to a much broader group of strains that usually share at least 95% of MIRU-VNTR pattern similarity: the Lisboa family [7]. One of the future goals is to better understand the populational structure of this family of strains, from which the Lisboa3 clade has differentiated, as strains belonging to this family have previously shown the potential to evolve to MDR-TB [4].

Another important point coming from the present student is the utility of WGS for epidemiological surveillance and strain typing. WGS presents an advantage over the classical typing methods (RFLP-IS6110, Spoligotyping or MIRU-VNTR) as it enables picturing transmission at a much higher resolution and ascertain isolate relatedness using well described models of molecular evolution. In the present study, WGS allowed strain discrimination within MIRU-VNTR clusters and distinguish between three independent Lisboa3 MDR sub-clades. Despite the technical difficulties in data analysis, as WGS costs converge towards the cost of MIRU-VNTR, the former is likely to replace MIRU-VNTR as the gold-standard for molecular epidemiological surveillance and strain typing. WGS can also enable more focused contact tracing by reducing the number of plausible genomically linked cases to investigate, leading to an improved case detection. WGS-assisted routine surveillance is still far away for many settings, but as this technology becomes gradually available to the mycobacteriology laboratory it will also be expected a greater understanding of TB transmission. In a recent study, Walker et al. have defined a threshold of 12 SNPs of difference, above which recent transmission can be excluded [75]. In our study, the number of unique SNPs to each isolate determined for the Lisboa3 and Q1 clades are consistent with ongoing recent transmission. This finding allied with the genomic uniqueness of these strains are of special importance not only locally but in a macro-epidemiological context. It is likely that these strains may spread to other parts of the world, due to increasing global travel and migratory waves, and be the cause of additional public health concern [76–78]. A recent report of an RD^RIO^ strain recovered from a remote location in Tibet alerts to this possibility [79].

It is also worth having in consideration that the host residing bacilli population has a certain degree of heterogeneity that can be overlooked through WGS but nonetheless lead to a higher than expected genomic difference after transmission. Similarly, selection during drug treatment might also artificially extend genomic distances. In this regard, classical genotyping using more stable markers might prove useful. The present study also stresses the need of further genomic studies in order to contribute to a *M. tuberculosis* genome-wide evolutive scenario, representative of different settings.

This, together with clinical data, will ultimately enable GWAS with a positive impact in TB management.

## Conclusions

In conclusion, it was found that the two main genetic clusters responsible for the great majority of MDR-TB in Portugal form two monophyletic clades (Lisboa3 and Q1) that denote sequential resistance amplification and/or independent resistance acquisition. The data supports the notion of ongoing MDR-TB transmission and endemicity associated with Lisboa3 and Q1 clades. The results obtained also support notion of a higher genomic diversity than the one usually associated with *M. tuberculosis*, mostly acquired through genome downsizing and non-synonymous SNPs. Different deletions were found to be specific to a number of lineages, of which some may carry functional consequences. Specifically, the 112 bp deletion on PPE41 gene that, found among Lisboa3 strains, may provide a selective advantage for these strains. Different SNP acquisition dynamics were also identified between the two clades which are suggestive of different adaptation strategies in which the transposition of IS*6110* may also have an important role in modulating gene expression and integrity.

## Methods

### Isolates and genetic data

The study consists of 56 *M. tuberculosis* clinical isolates (source: 55 Lisbon, 1 Oporto) recovered from laboratories and hospital units across Lisbon Health region. This set of isolates comprises a convenience sample of M. tuberculosis clinical isolates received for genotypic analysis at the Mycobacteria Laboratory from the Faculty of Pharmacy of the University of Lisbon. The sample is composed by drug resistant isolates plus additional susceptible isolates found to be genetically close (MIRU-VNTR) to the drug resistant isolates. All isolates underwent drug susceptibility testing for INH, RIF, STP, EMB and PZA and second-line drugs using standard methods (see [4]). DNA extraction was performed from culture growth on Lowenstein-Jensen medium slants using the cetyl trimethylammonium bromide methodology [80]. The DNA was used in genotyping by the 24-*loci* MIRU-VNTR method (see previous work, [81]). Extracted DNA was also subjected to whole-genome (101 bp paired end) sequencing at the KAUST genomics facility using the Illumina HiSeq 2000 platform (500 bp insert size). We also complemented this data using sequences in the public domain (F11, CDC1551, KZN1435, KZN4207, KZN605, KZN_R506, KZN_V2475, UT205 RGTB327, RGTB423, CCDC5180, CCDC5079, CTRI-2, BTB05_552, BTB05_559, S96_129, HN878, R1207, and X122 (all from the NCBI database).

### Genomic variant detection

The raw Illumina sequencing data was aligned to the H37Rv reference genome using the Burrows-Wheeler Alignment Tool v.0.6.1, yielding high coverage data for all isolates (mean read depth per position, mean 249.9, range 44–1411 fold; mean 99.1% genome covered, range 98.6 - 99.9%) (Table 1) [82]. Single nucleotide polymorphisms (SNPs) and small indels (<30 bp) were called using SAMtools software (v0.1.18) [83]. Other small indels (<100 bp) were detected using the software Pindel [84]. Only variants supported by at least ten sequence reads were considered. Detection of larger structural variants was performed using the SVMerge v1.2 pipeline combining Pindel v0.2.4 t, BreakDancer v1.1 Cpp package and, SECluster analysis outputs [85, 86]. Structural variant detection was done for each isolate alone and validation was achieved using comparison with local *de novo* assembly using Velvet [87]. *Loci* reported to be associated with regional differences [22, 23, 88] were identified using the alignments and coverage.

For insertion sequence (IS) mapping, reads containing specific oligonucleotide sequence of both 5′ and 3′ extremities (listed in Additional file 22) were extracted and, flanking genomic regions of interest concatenated in FASTA format producing one file for each extremity for each strain. Local BLAST analysis (standalone NCBI BLAST v.2.2.27+) was carried out for each file against *M. tuberculosis* H37Rv reference genome, minimum supporting read depth used to as a quality filter (10 for isolates with >500 fold coverage, 2 for the remaining). For IS*6110* BLAST hits, a mapping quality classification scheme was established consisting in high confidence, medium confidence and lesser confidence sites. Paired sites corresponding to mapping of both 5′ and 3′ ends in all isolates on which it occurred were classified as high confidence sites. Paired insertion sites for which both ends were mapped in at least 50% of the isolates on which they were found to occur were considered of medium confidence. Insertion sites in which only one end of the IS*6110* was mapped were considered of lesser confidence. Furthermore, insertion site hits mapped to *M. tuberculosis* H37Rv were excluded to avoid repetitive mapping.

### Other bioinformatics

The genomic data of publicly available *M. tuberculosis* strains (format FASTA) were included in the analysis through conversion to FASTQ format reads using the program dwgsim v.0.1.10, and mapped and analyzed as described above. When necessary, DNA sequence alignment was performed using the CLC Sequence Viewer v7.6.1 (CLC bio®, Aarhus N, Denmark) and visualized in BioEdit v7.1.3.0 (T. Hall).

A MIRU-VNTR-based dendrogram was constructed in the public MIRU-VNTR*plus* database using the D_sw_ measure of genetic distance for tandem repeat loci [89] and the Unweighted Pair Group Method with Arithmetic Averages (UPGMA) clustering method. Spoligotyping profile was inferred from raw read data using the SpolPred software followed by comparison to the SITVIT WEB database [40, 90]. A phylogenetic tree based on SNPs was constructed using Seaview 4.3.5 [91] using the Maximum Likelihood method. The analysis involved 76 nucleotide sequences with a total of 11271 sites in the final dataset. Tree topology was tested using the most recent approximate Likelihood Ratio Test (aLRT) as an alternative to bootstrap.

Putative impact of selected compensatory mutations on protein function was assessed through the use of SIFT scores (available at http://sift.jcvi.org/) [31] computed from the query alignment against UniRef90 database hits (with less than 90% identity, with a median sequence conservation equal to 3.00).

Any statistical analysis was conducted using the SPSS software.

### Data access

All sequencing data have been submitted to the European Nucleotide Archive (http://www.ebi.ac.uk/ena/) under study accession number ERP002611. Phylogenetic data (tree and alignment matrix) have been submitted to TreeBase under Study ID no. 16158 (URL: http://treebase.org/treebase-web/home.html).

## Electronic supplementary material

Additional file 1: **Boxplot graph showing the different types of SNP mutations.** (PDF 12 KB)

Additional file 2: **Distribution of RD deletions found across the analyzed genomes of 75 M. tuberculosis isolates.** RD absence is assigned with a black square and, Lisboa3 and Q1 clade isolates are highlighted in red and blue, respectively. Column and line totals account for the total number of RDs in column or line, respectively. (XLSX 312 KB)

Additional file 3: **Structural variability among sequenced strains.** (PDF 140 KB)

Additional file 4: **List of short deletions (<100 bp) found among the group of 75 clinical isolates.** Black squares indicate deletion detection. MIRU-VNTR cluster indicates the 24-loci MIRU-VNTR cluster of any given isolate, except if non-clustered (NC) or not determined (nd). Line and column totals indicate total column/line count of deletions. Isolates highlighted in red and blue belong to Lisboa3 and Q1 clade, respectively. (XLSX 609 KB)

Additional file 5: **List of short insertions (<100 bp) found among the group of 75 clinical isolates.** Black squares indicate insertion detection. MIRU-VNTR cluster indicates the 24-loci MIRU-VNTR cluster of any given isolate, except if non-clustered (NC) or not determined (nd). Line and column totals indicate total column/line count of insertions. Isolates highlighted in red and blue belong to Lisboa3 and Q1 clade, respectively. (XLSX 1 MB)

Additional file 6: **List of selected clade-defining candidate SVs, its position, size and affected ORFs.** Each clade-defining candidate SV was selected based on phylogenetic congruence and presence in all members of the specified clade. (XLSX 12 KB)

Additional file 7: **Types and number of large SVs (≥100 bp) found among the 75 analyzed isolates using the SVMerge pipeline and local assembly validation.** (XLSX 9 KB)

Additional file 8: **List of SV types found amoing the 75 clinical isolates group using the SVMerge pipeline and excluding copy number gain hits.** SV type includes: deletions (DEL); completely (INSi) and incompletely (INS) reconstructed insertions; simple inversions (INV) and complex inversions (INVCOMPLEX); deletions plus insertions (DELINS); and, inversions plus deletions (INVDEL) or plus insertions (INVINS). Black squares are indicative of SV detection. MIRU-VNTR cluster indicates the 24-loci MIRU-VNTR cluster of any given isolate, except if non-clustered (NC) or not determined (nd). Line and column totals indicate total column/line count of SVs. Isolates highlighted in red and blue belong to Lisboa3 and Q1 clade, respectively. (XLSX 214 KB)

Additional file 9: **Number of mutations categorized by structural and functional effect type found along specified branches of the Lisboa3 subtree.** (XLSX 11 KB)

Additional file 10: **Number of mutations categorized by structural and functional effect type found along specified branches of the Q1 subtree.** (XLSX 10 KB)

Additional file 11: **Intra-clade SNP diversity and uniqueness. Number of SNPs unique to each isolate and percentage of total SNPs detected.** Represented below each clade designation are: the number of SNPs that represents the total pool of SNPs shared by all isolates belonging to the respective clade; and, the range of the total percentage that this latter SNP pool count comprises from the total percentage of the isolates’ detected SNPs. (PDF 268 KB)

Additional file 12: **Mutations found to be acquired along node-delimited branches in the Lisboa3 subtree.** Position, Reference Sequence and Mutated Sequence are derived from the VCF format. (XLSX 52 KB)

Additional file 13: **Mutations found to be acquired along node-delimited branches in the Q1 subtree.** Position, Reference Sequence and Mutated Sequence are derived from the VCF format. (XLSX 18 KB)

Additional file 14: **Molecular model of Escherichia coli core RNA polymerase (Opalka et al.** [55]**) (RCSB Protein Data Bank ref. 3 LU0) showing the homologous RpoC residues found to be involved in putative RIF resistance compensation in M. tuberculosis.** The different RNA polymerase subunits are shown: Alpha/RpoA (blue chain), Beta/RpoB (brown chain), Beta’/RpoC (green chain) and Omega/RpoZ (grey chain). The RpoC highlighted residues, in red, Gly367, Trp409 and Lys1251 are homologous to the RpoC residues Gly442, Trp484 and Lys1152 from M. tuberculosis, respectively. (PDF 302 KB)

Additional file 15: **Genomic mapping of Insertion Sequnces relative to the genome of M. tuberculosis H37Rv.** Black squares indicate presence of the IS at the specified locations by mapping of both 5′ and 3′ ends, if both ends were used in mapping analysis. MIRU-VNTR cluster indicates the 24-loci MIRU-VNTR cluster of any given isolate, except if non-clustered (NC) or not determined (nd). Grey quares indicate mapping of only one end. (XLSX 19 KB)

Additional file 16: **Mapped positions of IS6110 found across the genomes of the 75 analyzed M. tuberculosis clinical isolates in relation to M. tuberculosis H37Rv.** Each mapped position shown refers to a IS6110 end from which the genomic position of insertion was deduced, referred on the Mapped End column. Chain column shows the chain coding for IS6110 copy in question and consequently, its orientation. Confidence column corresponds to the quality/confidence level classification explained in the Materials and Methods section. ORF column shows: the affected ORF in case of an intragenic insertion site; intergenic if the site is intergenic and mapped IS is not on the proper orientation to exert a putative upregulatory effect on an ORF located downstream of the IS 3′ end; or, the preffix up indicating that the mapped IS is upstream and in the same orientation of a downstream ORF, followed by a number indicating the distance to the downstream ORF and, followed by the ORF designation, gene or feature designation. Black squares indicate IS6110 copies mapped at both 5′ and 3′ end; grey squares indicate IS6110 copies that only the mapped end indicated in the Mapped End column was mapped; and, yellow squares indicate IS6110 copies on which the only mapped end is the other end than the one indicated in the Mapped End column. MIRU-VNTR cluster indicates the 24-loci MIRU-VNTR cluster of any given isolate, except if non-clustered (NC) or not determined (nd). Column and line totals account for the number of IS6110 copies mapped on each line and column, respectively. Isolates highlighted in red and blue belong to Lisboa3 and Q1 clades, respectively. (XLSX 85 KB)

Additional file 17: **Multiple comparison test results upon comparison of mean overall Ns/S and Tv/Ts ratios for four groups of strains: Lisboa3, Q1, Beijing clades and, other non clustered strains (NC).** Significant differences at the 0.05 level are highlighted in bold. (XLSX 12 KB)

Additional file 18: **Multiple comparison test results upon comparison of mean Ns/S and Tv/Ts ratios across the four genomic quadrants.** Significant differences at the 0.05 level are highlighted in bold. (XLSX 12 KB)

Additional file 19: **Multiple comparison test results upon comparison of mean Ns/S and Tv/Ts ratios across the four genomic quadrants for four groups of strains: Lisboa3, Q1, Beijing clades and, other non clustered strains (NC).** Significant differences at the 0.05 level are highlighted in bold. (XLSX 14 KB)

Additional file 20: **Multiple comparison test results upon comparison of mean Ns/S ratios across the different COGs for four groups of strains: Lisboa3, Q1, Beijing clades and, other non clustered strains (NC).** Significant differences at the 0.05 level are highlighted in bold. (XLSX 20 KB)

Additional file 21: **Multiple comparison test results upon comparison of mean Tv/Ts ratios across the different COGs for four groups of strains: Lisboa3, Q1, Beijing clades and, other non clustered strains (NC).** Significant differences at the 0.05 level are highlighted in bold. (XLSX 19 KB)

Additional file 22: **End sequences from the different ISs used as probes to extract reads for mapping analysis.** (XLSX 10 KB)

## Abbreviations

AMK: Amikacin
CAP: Capreomycin
EMB: Ethambutol
XDR: Extensive drug resistance
FQ: Fluoroquinolone
GWAS: Genome-wide association studies
IS: Insertion sequence
INH: Isoniazid
MDR: Multidrug resistance
MIRU: Mycobacterial interspersed repetitive unit
*M. tuberculosis*: *Mycobacterium tuberculosis*
N_s_: Non-synonymous
PGG: Principal genetic group
RD: Region of difference
RFLP: Restriction fragment length polymorphism
RIF: Rifampicin
SIT: Shared international type
SNP: Single nucleotide polymorphism
SCG: SNP cluster group
S: Synonymous
T_s_: Transition
T_v_: Transversion
TB: Tuberculosis
VNTR: Variable number of tandem repeats
WHO: World health organization.

## References

[1] European Centre for Disease Prevention and Control/WHO Regional Office for Europe (2012). Tuberculosis surveillance and monitoring in Europe 2012.

[2] World Health Organization (2012). Global Tuberculosis Control 2012.

[3] Abubakar I, Zignol M, Falzon D, Raviglione MC, Ditiu L, Masham S, Adetifa I, Ford N, Cox H, Lawn SD, Marais BJ, McHugh TD, Mwaba P, Bates M, Lipman M, Zijenah L, Logan S, McNerney R, Zumla A, Sarda K, Nahid P, Hoelscher M, Pletschette M, Memish ZA, Kim P, Hafner R, Cole S, Migliori GB, Maeurer M, Schito M (2013). Drug-resistant tuberculosis: time for a visionary political leadership. Lancet Infect Dis.

[4] Perdigao J, Macedo R, Joao I, Fernandes E, Brum L, Portugal I (2008). Multidrug-resistant tuberculosis in Lisbon, Portugal: a molecular epidemiological perspective. Microb Drug Resist.

[5] Perdigao J, Macedo R, Malaquias A, Ferreira A, Brum L, Portugal I (2010). Genetic analysis of extensively drug-resistant Mycobacterium tuberculosis strains in Lisbon, Portugal. J Antimicrob Chemother.

[6] Perdigao J, Macedo R, Silva C, Machado D, Couto I, Viveiros M, Jordao L, Portugal I (2013). From multidrug-resistant to extensively drug-resistant tuberculosis in Lisbon, Portugal: the stepwise mode of resistance acquisition. J Antimicrob Chemother.

[7] Portugal I, Covas MJ, Brum L, Viveiros M, Ferrinho P, Moniz-Pereira J, David H (1999). Outbreak of multiple drug-resistant tuberculosis in Lisbon: detection by restriction fragment length polymorphism analysis. Int J Tuberc Lung Dis.

[8] Portugal I, Maia S, Moniz-Pereira J (1999). Discrimination of multidrug-resistant Mycobacterium tuberculosis IS6110 fingerprint subclusters by rpoB gene mutation analysis. J Clin Microbiol.

[9] Perdigao J, Macedo R, Silva C, Pinto C, Furtado C, Brum L, Portugal I (2011). Tuberculosis drug-resistance in Lisbon, Portugal: a 6-year overview. Clin Microbiol Infect.

[10] Perdigao J, Macedo R, Machado D, Silva C, Jordao L, Couto I, Viveiros M, Portugal I (2014). GidB mutation as a phylogenetic marker for Q1 cluster Mycobacterium tuberculosis isolates and intermediate-level streptomycin resistance determinant in Lisbon. Portugal Clin Microbiol Infect.

[11] Gagneux S, Small PM (2007). Global phylogeography of Mycobacterium tuberculosis and implications for tuberculosis product development. Lancet Infect Dis.

[12] Hershberg R, Lipatov M, Small PM, Sheffer H, Niemann S, Homolka S, Roach JC, Kremer K, Petrov DA, Feldman MW, Gagneux S (2008). High functional diversity in Mycobacterium tuberculosis driven by genetic drift and human demography. PLoS Biol.

[13] Niemann S, Koser CU, Gagneux S, Plinke C, Homolka S, Bignell H, Carter RJ, Cheetham RK, Cox A, Gormley NA, Kokko-Gonzales P, Murray LJ, Rigatti R, Smith VP, Arends FP, Cox HS, Smith G, Archer JA (2009). Genomic diversity among drug sensitive and multidrug resistant isolates of Mycobacterium tuberculosis with identical DNA fingerprints. PLoS One.

[14] Sreevatsan S, Pan X, Stockbauer KE, Connell ND, Kreiswirth BN, Whittam TS, Musser JM (1997). Restricted structural gene polymorphism in the Mycobacterium tuberculosis complex indicates evolutionarily recent global dissemination. Proc Natl Acad Sci U S A.

[15] Ioerger TR, Feng Y, Ganesula K, Chen X, Dobos KM, Fortune S, Jacobs WR, Mizrahi V, Parish T, Rubin E, Sassetti C, Sacchettini JC (2010). Variation among genome sequences of H37Rv strains of Mycobacterium tuberculosis from multiple laboratories. J Bacteriol.

[16] Ford C, Yusim K, Ioerger T, Feng S, Chase M, Greene M, Korber B, Fortune S (2012). Mycobacterium tuberculosis--heterogeneity revealed through whole genome sequencing. Tuberculosis (Edinburgh, Scotland).

[17] Schurch AC, Kremer K, Kiers A, Daviena O, Boeree MJ, Siezen RJ, Smith NH, van Soolingen D (2009). The tempo and mode of molecular evolution of Mycobacterium tuberculosis at patient-to-patient scale. Infect Genet Evol.

[18] Casali N, Nikolayevskyy V, Balabanova Y, Ignatyeva O, Kontsevaya I, Harris SR, Bentley SD, Parkhill J, Nejentsev S, Hoffner SE, Horstmann RD, Brown T, Drobniewski F (2012). Microevolution of extensively drug-resistant tuberculosis in Russia. Genome Res.

[19] Ioerger TR, Koo S, No EG, Chen X, Larsen MH, Jacobs WR, Pillay M, Sturm AW, Sacchettini JC (2009). Genome analysis of multi- and extensively-drug-resistant tuberculosis from KwaZulu-Natal. South Africa PLoS One.

[20] Filliol I, Motiwala AS, Cavatore M, Qi W, Hazbon MH, Bobadilla del Valle M, Fyfe J, Garcia-Garcia L, Rastogi N, Sola C, Zozio T, Guerrero MI, Leon CI, Crabtree J, Angiuoli S, Eisenach KD, Durmaz R, Joloba ML, Rendon A, Sifuentes-Osornio J, Ponce De Leon A, Cave MD, Fleischmann R, Whittam TS, Alland D (2006). Global phylogeny of Mycobacterium tuberculosis based on single nucleotide polymorphism (SNP) analysis: insights into tuberculosis evolution, phylogenetic accuracy of other DNA fingerprinting systems, and recommendations for a minimal standard SNP set. J Bacteriol.

[21] Lazzarini LC, Huard RC, Boechat NL, Gomes HM, Oelemann MC, Kurepina N, Shashkina E, Mello FC, Gibson AL, Virginio MJ, Marsico AG, Butler WR, Kreiswirth BN, Suffys PN, Lapa ESJR, Ho JL (2007). Discovery of a novel Mycobacterium tuberculosis lineage that is a major cause of tuberculosis in Rio de Janeiro, Brazil. J Clin Microbiol.

[22] Tsolaki AG, Hirsh AE, DeRiemer K, Enciso JA, Wong MZ, Hannan M, Goguet de la Salmoniere YO, Aman K, Kato-Maeda M, Small PM (2004). Functional and evolutionary genomics of Mycobacterium tuberculosis: insights from genomic deletions in 100 strains. Proc Natl Acad Sci U S A.

[23] Gagneux S, DeRiemer K, Van T, Kato-Maeda M, de Jong BC, Narayanan S, Nicol M, Niemann S, Kremer K, Gutierrez MC, Hilty M, Hopewell PC, Small PM (2006). Variable host-pathogen compatibility in Mycobacterium tuberculosis. Proc Natl Acad Sci U S A.

[24] Gibson AL, Huard RC, Gey van Pittius NC, Lazzarini LC, Driscoll J, Kurepina N, Zozio T, Sola C, Spindola SM, Kritski AL, Fitzgerald D, Kremer K, Mardassi H, Chitale P, Brinkworth J, Garcia de Viedma D, Gicquel B, Pape JW, van Soolingen D, Kreiswirth BN, Warren RM, Van Helden PD, Rastogi N, Suffys PN, Lapa e Silva J, Ho JL (2008). Application of sensitive and specific molecular methods to uncover global dissemination of the major RDRio Sublineage of the Latin American-Mediterranean Mycobacterium tuberculosis spoligotype family. J Clin Microbiol.

[25] Madhavilatha GK, Joseph BV, Paul LK, Kumar RA, Hariharan R, Mundayoor S (2012). Whole-genome sequences of two clinical isolates of Mycobacterium tuberculosis from Kerala, South India. J Bacteriol.

[26] Srivastava S, Garg A, Ayyagari A, Nyati KK, Dhole TN, Dwivedi SK (2006). Nucleotide polymorphism associated with ethambutol resistance in clinical isolates of Mycobacterium tuberculosis. Curr Microbiol.

[27] Machado D, Perdigao J, Ramos J, Couto I, Portugal I, Ritter C, Boettger EC, Viveiros M (2013). High-level resistance to isoniazid and ethionamide in multidrug-resistant Mycobacterium tuberculosis of the Lisboa family is associated with inhA double mutations. J Antimicrob Chemother.

[28] Gagneux S, Long CD, Small PM, Van T, Schoolnik GK, Bohannan BJ (2006). The competitive cost of antibiotic resistance in Mycobacterium tuberculosis. Science (New York, NY).

[29] Comas I, Borrell S, Roetzer A, Rose G, Malla B, Kato-Maeda M, Galagan J, Niemann S, Gagneux S (2012). Whole-genome sequencing of rifampicin-resistant Mycobacterium tuberculosis strains identifies compensatory mutations in RNA polymerase genes. Nat Genet.

[30] de Vos M, Muller B, Borrell S, Black P, van Helden P, Warren R, Gagneux S, Victor T (2012). Putative compensatory mutations in the rpoC gene of rifampicin-resistant Mycobacterium tuberculosis are associated with ongoing transmission. Antimicrob Agents Chemother.

[31] Kumar P, Henikoff S, Ng PC (2009). Predicting the effects of coding non-synonymous variants on protein function using the SIFT algorithm. Nat Protoc.

[32] Casart Y, Turcios L, Florez I, Jaspe R, Guerrero E, de Waard J, Aguilar D, Hernandez-Pando R, Salazar L (2008). IS6110 in oriC affects the morphology and growth of Mycobacterium tuberculosis and attenuates virulence in mice. Tuberculosis (Edinburgh, Scotland).

[33] Soto CY, Menendez MC, Perez E, Samper S, Gomez AB, Garcia MJ, Martin C (2004). IS6110 mediates increased transcription of the phoP virulence gene in a multidrug-resistant clinical isolate responsible for tuberculosis outbreaks. J Clin Microbiol.

[34] Kurepina N, Likhoshvay E, Shashkina E, Mathema B, Kremer K, van Soolingen D, Bifani P, Kreiswirth BN (2005). Targeted hybridization of IS6110 fingerprints identifies the W-Beijing Mycobacterium tuberculosis strains among clinical isolates. J Clin Microbiol.

[35] Plikaytis BB, Marden JL, Crawford JT, Woodley CL, Butler WR, Shinnick TM (1994). Multiplex PCR assay specific for the multidrug-resistant strain W of Mycobacterium tuberculosis. J Clin Microbiol.

[36] Namouchi A, Didelot X, Schock U, Gicquel B, Rocha EP (2012). After the bottleneck: Genome-wide diversification of the Mycobacterium tuberculosis complex by mutation, recombination, and natural selection. Genome Res.

[37] Alland D, Lacher DW, Hazbon MH, Motiwala AS, Qi W, Fleischmann RD, Whittam TS (2007). Role of large sequence polymorphisms (LSPs) in generating genomic diversity among clinical isolates of Mycobacterium tuberculosis and the utility of LSPs in phylogenetic analysis. J Clin Microbiol.

[38] Lin J, Sattar AN, Puckree T (2004). An alarming rate of drug-resistant tuberculosis at Ngwelezane Hospital in northern KwaZulu Natal, South Africa. Int J Tuberc Lung Dis.

[39] Pillay M, Sturm AW (2007). Evolution of the extensively drug-resistant F15/LAM4/KZN strain of Mycobacterium tuberculosis in KwaZulu-Natal, South Africa. Clin Infect Dis.

[40] Demay C, Liens B, Burguiere T, Hill V, Couvin D, Millet J, Mokrousov I, Sola C, Zozio T, Rastogi N (2012). SITVITWEB–a publicly available international multimarker database for studying Mycobacterium tuberculosis genetic diversity and molecular epidemiology. Infect Genet Evol.

[41] Lazzarini LC, Spindola SM, Bang H, Gibson AL, Weisenberg S, da Silva CW, Augusto CJ, Huard RC, Kritski AL, Ho JL (2008). RDRio Mycobacterium tuberculosis infection is associated with a higher frequency of cavitary pulmonary disease. J Clin Microbiol.

[42] Akhter Y, Ehebauer MT, Mukhopadhyay S, Hasnain SE (2012). The PE/PPE multigene family codes for virulence factors and is a possible source of mycobacterial antigenic variation: perhaps more?. Biochimie.

[43] Mukhopadhyay S, Balaji KN (2011). The PE and PPE proteins of Mycobacterium tuberculosis. Tuberculosis (Edinburgh, Scotland).

[44] Behr MA, Warren SA, Salamon H, Hopewell PC, Ponce de Leon A, Daley CL, Small PM (1999). Transmission of Mycobacterium tuberculosis from patients smear-negative for acid-fast bacilli. Lancet.

[45] Hernandez-Garduno E, Cook V, Kunimoto D, Elwood RK, Black WA, FitzGerald JM (2004). Transmission of tuberculosis from smear negative patients: a molecular epidemiology study. Thorax.

[46] Tostmann A, Kik SV, Kalisvaart NA, Sebek MM, Verver S, Boeree MJ, van Soolingen D (2008). Tuberculosis transmission by patients with smear-negative pulmonary tuberculosis in a large cohort in the Netherlands. Clin Infect Dis.

[47] Choudhary RK, Mukhopadhyay S, Chakhaiyar P, Sharma N, Murthy KJ, Katoch VM, Hasnain SE (2003). PPE antigen Rv2430c of Mycobacterium tuberculosis induces a strong B-cell response. Infect Immun.

[48] Tundup S, Pathak N, Ramanadham M, Mukhopadhyay S, Murthy KJ, Ehtesham NZ, Hasnain SE (2008). The co-operonic PE25/PPE41 protein complex of Mycobacterium tuberculosis elicits increased humoral and cell mediated immune response. PLoS One.

[49] Fenner L, Egger M, Bodmer T, Altpeter E, Zwahlen M, Jaton K, Pfyffer GE, Borrell S, Dubuis O, Bruderer T, Siegrist HH, Furrer H, Calmy A, Fehr J, Stalder JM, Ninet B, Bottger EC, Gagneux S (2012). Effect of mutation and genetic background on drug resistance in Mycobacterium tuberculosis. Antimicrob Agents Chemother.

[50] Brimacombe M, Hazbon M, Motiwala AS, Alland D (2007). Antibiotic resistance and single-nucleotide polymorphism cluster grouping type in a multinational sample of resistant Mycobacterium tuberculosis isolates. Antimicrob Agents Chemother.

[51] Maus CE, Plikaytis BB, Shinnick TM (2005). Molecular analysis of cross-resistance to capreomycin, kanamycin, amikacin, and viomycin in Mycobacterium tuberculosis. Antimicrob Agents Chemother.

[52] Richardson ET, Lin SY, Pinsky BA, Desmond E, Banaei N (2009). First documentation of isoniazid reversion in Mycobacterium tuberculosis. Int J Tuberc Lung Dis.

[53] Brandis G, Wrande M, Liljas L, Hughes D (2012). Fitness-compensatory mutations in rifampicin-resistant RNA polymerase. Mol Microbiol.

[54] Farhat MR, Shapiro BJ, Kieser KJ, Sultana R, Jacobson KR, Victor TC, Warren RM, Streicher EM, Calver A, Sloutsky A, Kaur D, Posey JE, Plikaytis B, Oggioni MR, Gardy JL, Johnston JC, Rodrigues M, Tang PK, Kato-Maeda M, Borowsky ML, Muddukrishna B, Kreiswirth BN, Kurepina N, Galagan J, Gagneux S, Birren B, Rubin EJ, Lander ES, Sabeti PC, Murray M (2013). Genomic analysis identifies targets of convergent positive selection in drug-resistant Mycobacterium tuberculosis. Nat Genet.

[55] Opalka N, Brown J, Lane WJ, Twist KA, Landick R, Asturias FJ, Darst SA (2010). Complete structural model of Escherichia coli RNA polymerase from a hybrid approach. PLoS Biol.

[56] Heep M, Brandstatter B, Rieger U, Lehn N, Richter E, Rusch-Gerdes S, Niemann S (2001). Frequency of rpoB mutations inside and outside the cluster I region in rifampin-resistant clinical Mycobacterium tuberculosis isolates. J Clin Microbiol.

[57] Siu GK, Zhang Y, Lau TC, Lau RW, Ho PL, Yew WW, Tsui SK, Cheng VC, Yuen KY, Yam WC (2011). Mutations outside the rifampicin resistance-determining region associated with rifampicin resistance in Mycobacterium tuberculosis. J Antimicrob Chemother.

[58] Sherman DR, Mdluli K, Hickey MJ, Arain TM, Morris SL, Barry CE, Stover CK (1996). Compensatory ahpC gene expression in isoniazid-resistant Mycobacterium tuberculosis. Science (New York, NY).

[59] Shcherbakov D, Akbergenov R, Matt T, Sander P, Andersson DI, Bottger EC (2010). Directed mutagenesis of Mycobacterium smegmatis 16S rRNA to reconstruct the in-vivo evolution of aminoglycoside resistance in Mycobacterium tuberculosis. Mol Microbiol.

[60] Gagneux S, Burgos MV, DeRiemer K, Encisco A, Munoz S, Hopewell PC, Small PM, Pym AS (2006). Impact of bacterial genetics on the transmission of isoniazid-resistant Mycobacterium tuberculosis. PLoS Pathog.

[61] Safi H, Lingaraju S, Amin A, Kim S, Jones M, Holmes M, McNeil M, Peterson SN, Chatterjee D, Fleischmann R, Alland D (2013). Evolution of high-level ethambutol-resistant tuberculosis through interacting mutations in decaprenylphosphoryl-beta-D-arabinose biosynthetic and utilization pathway genes. Nat Genet.

[62] Zhang H, Li D, Zhao L, Fleming J, Lin N, Wang T, Liu Z, Li C, Galwey N, Deng J, Zhou Y, Zhu Y, Gao Y, Wang S, Huang Y, Wang M, Zhong Q, Zhou L, Chen T, Zhou J, Yang R, Zhu G, Hang H, Zhang J, Li F, Wan K, Wang J, Zhang XE, Bi L (2013). Genome sequencing of 161 Mycobacterium tuberculosis isolates from China identifies genes and intergenic regions associated with drug resistance. Nat Genet.

[63] de Boer AS, Borgdorff MW, de Haas PE, Nagelkerke NJ, van Embden JD, van Soolingen D (1999). Analysis of rate of change of IS6110 RFLP patterns of Mycobacterium tuberculosis based on serial patient isolates. J Infect Dis.

[64] Yeh RW, Ponce de Leon A, Agasino CB, Hahn JA, Daley CL, Hopewell PC, Small PM (1998). Stability of Mycobacterium tuberculosis DNA genotypes. J Infect Dis.

[65] Sampson S, Warren R, Richardson M, van der Spuy G, van Helden P (2001). IS6110 insertions in Mycobacterium tuberculosis: predominantly into coding regions. J Clin Microbiol.

[66] Cole ST, Brosch R, Parkhill J, Garnier T, Churcher C, Harris D, Gordon SV, Eiglmeier K, Gas S, Barry CE, Tekaia F, Badcock K, Basham D, Brown D, Chillingworth T, Connor R, Davies R, Devlin K, Feltwell T, Gentles S, Hamlin N, Holroyd S, Hornsby T, Jagels K, Krogh A, McLean J, Moule S, Murphy L, Oliver K, Osborne J (1998). Deciphering the biology of Mycobacterium tuberculosis from the complete genome sequence. Nature.

[67] Tanaka MM, Rosenberg NA, Small PM (2004). The control of copy number of IS6110 in Mycobacterium tuberculosis. Mol Biol Evol.

[68] Safi H, Barnes PF, Lakey DL, Shams H, Samten B, Vankayalapati R, Howard ST (2004). IS6110 functions as a mobile, monocyte-activated promoter in Mycobacterium tuberculosis. Mol Microbiol.

[69] Thorne N, Borrell S, Evans J, Magee J, Garcia de Viedma D, Bishop C, Gonzalez-Martin J, Gharbia S, Arnold C (2011). IS6110-based global phylogeny of Mycobacterium tuberculosis. Infect Genet Evol.

[70] Beste DJ, Hooper T, Stewart G, Bonde B, Avignone-Rossa C, Bushell ME, Wheeler P, Klamt S, Kierzek AM, McFadden J (2007). GSMN-TB: a web-based genome-scale network model of Mycobacterium tuberculosis metabolism. Genome Biol.

[71] Fang X, Wallqvist A, Reifman J (2010). Development and analysis of an in vivo-compatible metabolic network of Mycobacterium tuberculosis. BMC Syst Biol.

[72] Jamshidi N, Palsson BO (2007). Investigating the metabolic capabilities of Mycobacterium tuberculosis H37Rv using the in silico strain iNJ661 and proposing alternative drug targets. BMC Syst Biol.

[73] Hirsh AE, Tsolaki AG, DeRiemer K, Feldman MW, Small PM (2004). Stable association between strains of Mycobacterium tuberculosis and their human host populations. Proc Natl Acad Sci U S A.

[74] Jang J, Becq J, Gicquel B, Deschavanne P, Neyrolles O (2008). Horizontally acquired genomic islands in the tubercle bacilli. Trends Microbiol.

[75] Walker TM, Ip CL, Harrell RH, Evans JT, Kapatai G, Dedicoat MJ, Eyre DW, Wilson DJ, Hawkey PM, Crook DW, Parkhill J, Harris D, Walker AS, Bowden R, Monk P, Smith EG, Peto TE (2013). Whole-genome sequencing to delineate Mycobacterium tuberculosis outbreaks: a retrospective observational study. Lancet Infect Dis.

[76] Liu Y, Painter JA, Posey DL, Cain KP, Weinberg MS, Maloney SA, Ortega LS, Cetron MS (2012). Estimating the impact of newly arrived foreign-born persons on tuberculosis in the United States. PLoS One.

[77] Mor Z, Pinsker G, Cedar N, Lidji M, Grotto I (2012). Adult tuberculosis in Israel and migration: trends and challenges between 1999 and 2010. Int J Tuberc Lung Dis.

[78] Field V, Gautret P, Schlagenhauf P, Burchard GD, Caumes E, Jensenius M, Castelli F, Gkrania-Klotsas E, Weld L, Lopez-Velez R, de Vries P, von Sonnenburg F, Loutan L, Parola P (2010). Travel and migration associated infectious diseases morbidity in Europe, 2008. BMC Infect Dis.

[79] Mokrousov I, Jiao WW, Wan K, Shen A (2014). Stranger in a strange land: Ibero-American strain of Mycobacterium tuberculosis in Tibet, China. Infect Genet Evol.

[80] van Soolingen D, de Haas PEW, Kremer K (2002). Restriction fragment length polymorphism (RFLP) typing of mycobacteria. Bilthoven, The Netherlands: National Intitute of Public Health and The Environment.

[81] Supply P, Allix C, Lesjean S, Cardoso-Oelemann M, Rusch-Gerdes S, Willery E, Savine E, de Haas P, van Deutekom H, Roring S, Bifani P, Kurepina N, Kreiswirth B, Sola C, Rastogi N, Vatin V, Gutierrez MC, Fauville M, Niemann S, Skuce R, Kremer K, Locht C, van Soolingen D (2006). Proposal for standardization of optimized mycobacterial interspersed repetitive unit-variable-number tandem repeat typing of Mycobacterium tuberculosis. J Clin Microbiol.

[82] Li H, Durbin R (2009). Fast and accurate short read alignment with Burrows-Wheeler transform. Bioinformatics.

[83] Li H, Handsaker B, Wysoker A, Fennell T, Ruan J, Homer N, Marth G, Abecasis G, Durbin R (2009). The Sequence Alignment/Map format and SAMtools. Bioinformatics.

[84] Ye K, Schulz MH, Long Q, Apweiler R, Ning Z (2009). Pindel: a pattern growth approach to detect break points of large deletions and medium sized insertions from paired-end short reads. Bioinformatics.

[85] Chen K, Wallis JW, McLellan MD, Larson DE, Kalicki JM, Pohl CS, McGrath SD, Wendl MC, Zhang Q, Locke DP, Shi X, Fulton RS, Ley TJ, Wilson RK, Ding L, Mardis ER (2009). BreakDancer: an algorithm for high-resolution mapping of genomic structural variation. Nat Methods.

[86] Wong K, Keane TM, Stalker J, Adams DJ (2010). Enhanced structural variant and breakpoint detection using SVMerge by integration of multiple detection methods and local assembly. Genome Biol.

[87] Zerbino DR, Birney E (2008). Velvet: algorithms for de novo short read assembly using de Bruijn graphs. Genome Res.

[88] Brosch R, Gordon SV, Marmiesse M, Brodin P, Buchrieser C, Eiglmeier K, Garnier T, Gutierrez C, Hewinson G, Kremer K, Parsons LM, Pym AS, Samper S, van Soolingen D, Cole ST (2002). A new evolutionary scenario for the Mycobacterium tuberculosis complex. Proc Natl Acad Sci U S A.

[89] Shriver MD, Jin L, Boerwinkle E, Deka R, Ferrell RE, Chakraborty R (1995). A novel measure of genetic distance for highly polymorphic tandem repeat loci. Mol Biol Evol.

[90] Coll F, Mallard K, Preston MD, Bentley S, Parkhill J, McNerney R, Martin N, Clark TG (2012). SpolPred: rapid and accurate prediction of Mycobacterium tuberculosis spoligotypes from short genomic sequences. Bioinformatics.

[91] Gouy M, Guindon S, Gascuel O (2010). SeaView version 4: a multiplatform graphical user interface for sequence alignment and phylogenetic tree building. Mol Biol Evol.

