# Supplementary figures and images for: Unraveling Mycobacterium tuberculosis genomic diversity and evolution in Lisbon, Portugal, a highly drug resistant setting
Source: BMC Genomics. 2014 Nov 18;15(1):991. doi: 10.1186/1471-2164-15-991 (PMC4289236; doi:10.1186/1471-2164-15-991)

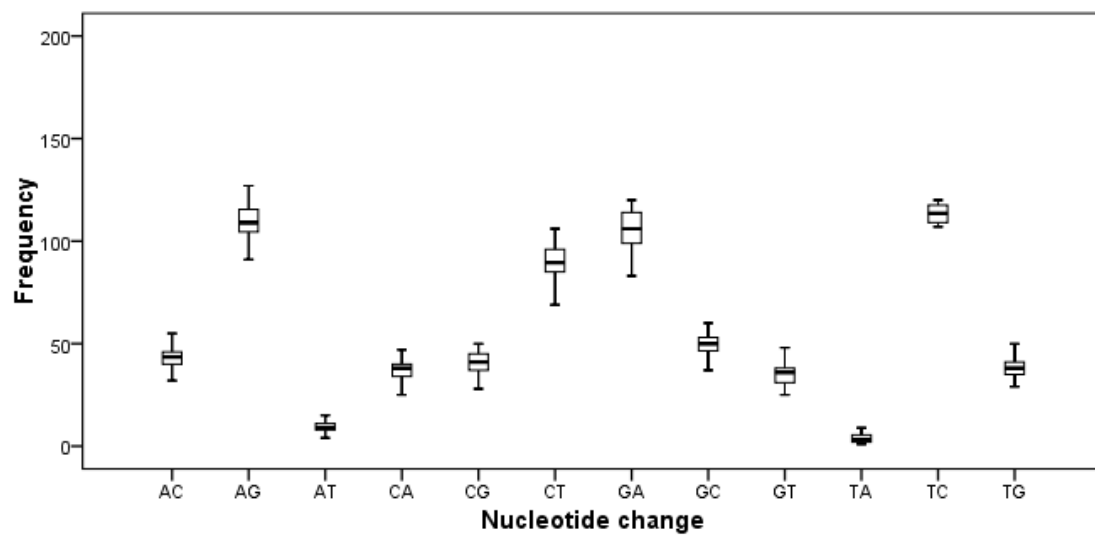

**Additional file 1** - Boxplot graph showing the different types of SNP mutations.

Supplement: Supplementary file 1 — Additional file 1: Boxplot graph showing the different types of SNP mutations. (PDF 12 KB) [file 12864_2013_6861_MOESM1_ESM.pdf]
