# Supplementary material for: Unraveling Mycobacterium tuberculosis genomic diversity and evolution in Lisbon, Portugal, a highly drug resistant setting
Source: BMC Genomics. 2014 Nov 18;15(1):991. doi: 10.1186/1471-2164-15-991 (PMC4289236; doi:10.1186/1471-2164-15-991)
Supplement: Supplementary file 3 — Additional file 3: Structural variability among sequenced strains. (PDF 140 KB) [file 12864_2013_6861_MOESM3_ESM.pdf]

Additional File 3

Structural variability among sequenced strains

Using the sequence data, we attempted to detect structural variants, including small insertions and deletions (< 100 bp) using the Pindel program and larger variants (≥100 bp) using a combination of different methods compared with local assembly (SVMerge pipeline, Wong *et al*.). Among the group of 75 isolates, 2143 different candidate deletions (sizes 1-99bp) and 4091 different candidate insertions (sizes 1-79) were detected (see Additional files 4 and 5). We have selected high quality short indels that appear to be clade associated and phylogenetically conserved among all members of the clade (see Additional file 6). These variants may be clade-specific and could carry functional consequences that reflect host adaptation and selection.

Eight types of larger SVs were detected (see Additional file 7). Copy number gains were excluded due to a lack of robustness of the methods applied. We have found that putative SVs when detected in more than one isolate mostly present a phylogenetically incongruent distribution. The candidate structural variants found in the present study are reported (see Additional file 8), some are identical, but only have similar breakpoints due to uneven coverage.

REFERENCES

Wong K, Keane TM, Stalker J, Adams DJ: **Enhanced structural variant and breakpoint detection using SVMerge by integration of multiple detection methods and local assembly**. *Genome Biol* 2010, **11**(12):R128.
