# Supplementary material for: Unraveling Mycobacterium tuberculosis genomic diversity and evolution in Lisbon, Portugal, a highly drug resistant setting
Source: BMC Genomics. 2014 Nov 18;15(1):991. doi: 10.1186/1471-2164-15-991 (PMC4289236; doi:10.1186/1471-2164-15-991)
Supplement: Supplementary file 11 — Additional file 11: Intra-clade SNP diversity and uniqueness. Number of SNPs unique to each isolate and percentage of total SNPs detected. Represented below each clade designation are: the number of SNPs that represents the total pool of SNPs shared by all isolates belonging to the respective clade; and, the range of the total percentage that this latter SNP pool count comprises from the total percentage of the isolates’ detected SNPs. (PDF 268 KB) [file 12864_2013_6861_MOESM11_ESM.pdf]

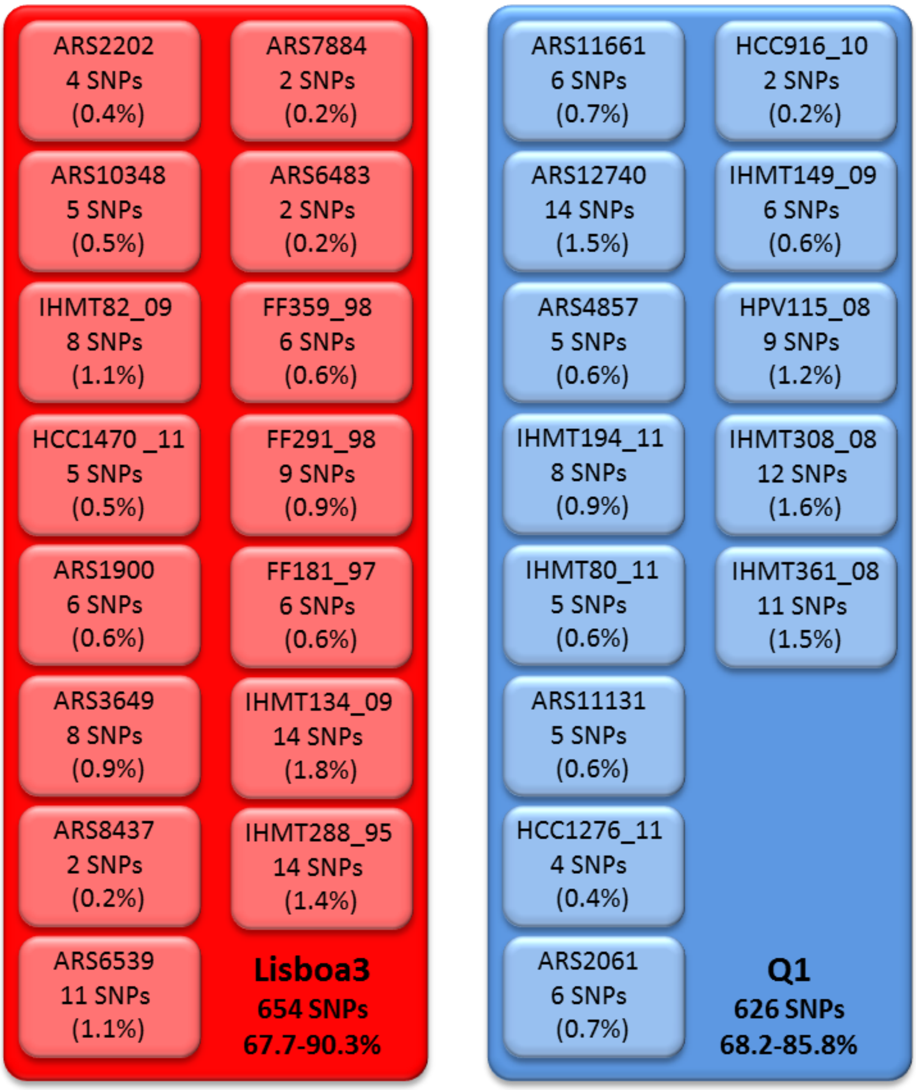

Additional file 11 – Intra-clade SNP diversity and uniqueness. Number of SNPs unique to each isolate and percentage of total SNPs detected. Represented below each clade designation are: the number of SNPs that represents the total pool of SNPs shared by all isolates belonging to the respective clade; and, the range of the total percentage that this latter SNP pool count comprises from the total percentage of the isolates' detected SNPs.
