# Supplementary material for: Unraveling Mycobacterium tuberculosis genomic diversity and evolution in Lisbon, Portugal, a highly drug resistant setting
Source: BMC Genomics. 2014 Nov 18;15(1):991. doi: 10.1186/1471-2164-15-991 (PMC4289236; doi:10.1186/1471-2164-15-991)
Supplement: Supplementary file 14 — Additional file 14: Molecular model of Escherichia coli core RNA polymerase (Opalka et al. [55]) (RCSB Protein Data Bank ref. 3 LU0) showing the homologous RpoC residues found to be involved in putative RIF resistance compensation in M. tuberculosis. The different RNA polymerase subunits are shown: Alpha/RpoA (blue chain), Beta/RpoB (brown chain), Beta’/RpoC (green chain) and Omega/RpoZ (grey chain). The RpoC highlighted residues, in red, Gly367, Trp409 and Lys1251 are homologous to the RpoC residues Gly442, Trp484 and Lys1152 from M. tuberculosis, respectively. (PDF 302 KB) [file 12864_2013_6861_MOESM14_ESM.pdf]

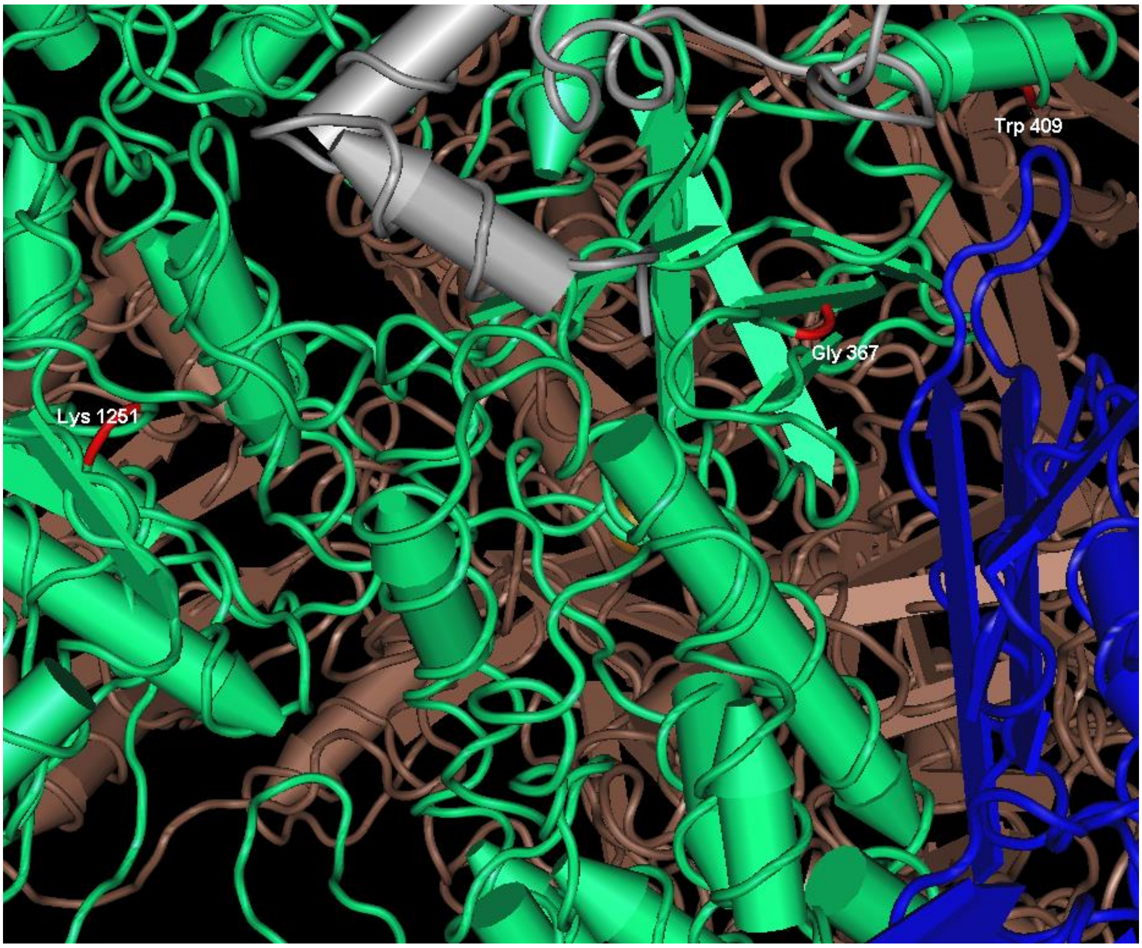

Additional file 14 – Molecular model of Escherichia coli core RNA polymerase (Opalka et al. 2010) (RCSB Protein Data Bank ref. 3LU0) showing the homologous RpoC residues found to be involved in putative RIF resistance compensation in *M. tuberculosis*. The different RNA polymerase subunits are shown: Alpha/RpoA (blue chain), Beta/RpoB (brown chain), Beta'/RpoC (green chain) and Omega/RpoZ (grey chain). The RpoC highlighted residues, in red, Gly367, Trp409 and Lys1251 are homologous to the RpoC residues Gly442, Trp484 and Lys1152 from *M. tuberculosis*, respectively.

REFERENCES

Opalka N, Brown J, Lane WJ, Twist KA, Landick R, Asturias FJ, Darst SA. 2010. Complete structural model of Escherichia coli RNA polymerase from a hybrid approach. *PLoS Biol* **8**(9).
